# The Oxidation Behavior of ZrB_2_-SiC Ceramic Composites Fabricated by Plasma Spray Process

**DOI:** 10.3390/ma14020392

**Published:** 2021-01-14

**Authors:** Eid M. Alosime, Mohammed S. Alsuhybani, Mohammed S. Almeataq

**Affiliations:** King Abdulaziz City for Science and Technology, Riyadh 11442, Saudi Arabia; alosimi@kacst.edu.sa (E.M.A.); sohybani@kacst.edu.sa (M.S.A.)

**Keywords:** silicon carbide, high-pressure plasma spray, oxidation resistance, Ultra-high temperature ceramic (UHTC)

## Abstract

Our goal is to develop a structural ceramic for high-temperature applications in which silicon carbide-based materials (SiCs) are used as matrix composites. The potential of SiCs to deposit a mixture of SiC and zirconium diboride (ZrB_2_) plasma spray coating is analyzed. To deposit thermal barrier layers containing up to 50 vol.% SiC, a high-pressure plasma spray (HPPS) process was used. Although the SiC cannot be deposited by thermal spray, a mixture of SiC and zirconium diboride (ZrB_2_) was deposited because these two compounds form a eutectic phase at a temperature below SiC decomposition. The preference was two different forms, 3 mm and 1 mm, of graphite substrates with different thickness values. A comparison of the morphology of SiC-ZrB_2_ coatings before and after thermal treatment was performed by applying heat to the surface of a gas torch and traditional furnace between 800 °C and 1200 °C. The growth of the oxide scale was calculated with X-ray diffraction (XRD), scanning electron microscopy (SEM)/energy dispersive X-ray analysis (EDX), transmission electron microscopy (TEM), and density. The oxide scale consists of a SiO_2_ layer with ZrO_2_ groups. The findings indicate a greater potential for the studied material in protecting against high-temperature oxidation and in a wide variety of aerospace applications.

## 1. Introduction

Because of their many uses, ultra-high-temperature ceramics (UHTCs) are considered highly significant, for example, for use in spacecraft applications and wall defense shields in nuclear reactors [1,2]. Indeed, ceramic materials with high chemical, mechanical, and thermal strength can be used at extremely high temperatures. In these applications, high temperatures and a highly reactive and flowing atmosphere (e.g., O, O_2_) are used for the materials. The potential candidates for the material must also be mechanically, chemically, and thermally stable.

Because of the importance and high demand of UHTC applications, research and development activities have increased in various ways to generate UHTCs. Temperatures ranging from 1000 to 2000 °C must be present for an UHTC to be functionally sound/stable [3,4]. There are three main classes commonly discussed in the applications for UHTCs. Superalloys have shown promising results in applications with ultra-high temperatures, but they have shown some restrictions regarding their usage in certain temperatures—working best at about 1400 °C to 1600 °C—which has spurred the search for an alternative. Furthermore, some ceramic materials have shown good features that meet the needs of these applications in thermal protection systems, but these materials have fallen short. Indeed, the need for the next generation of UHTCs has been motivated by low fractures and production difficulties. Ceramic matrix composites (CMCs) have shown promising properties for their application with high fracture resistance (over ceramic materials) and increased number of manufacturing techniques in these types of applications [5,6,7,8]. The use of CMCs is very successful. Furthermore, for high-temperature applications, carbon-carbon (C/C) composites have been used, but they oxidize at low temperatures of around 400 °C [9]. Additionally, for this type of application, previous trials have shown that the existence of dissociated gas molecules affects the microstructure and behavior of CMCs at high temperature [10,11]. Therefore, two fields of research have been identified: (i) composite carbon material for existing material replacement and (ii) composite carbon material for thermal protection [3] and thermal protective filing. The development of composites of refractory metal and other ceramics to produce a high-temperature (UHTCs) composite that acts as a thermal and oxidation barrier requires high-temperature applications between 0 °C and 2200 °C and reactive environments [12,13].

**Figure 1:**
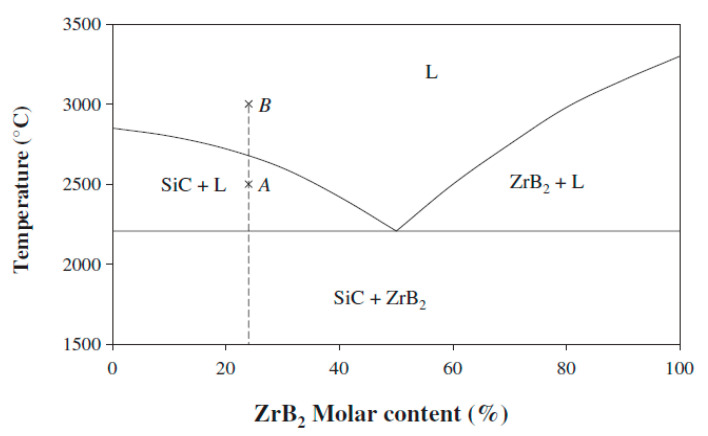
ZrB_2_-SiC phase diagram, where points *A* and *B* are particles impacted on the substrate in a completely melted status above liquidus (L) temperature [24].

A thermal barrier coating (TBC) is a state-of-the-art ceramic materials device that is deposited on top of a substrate such as metal and carbon fibers to boost their thermal isolation characteristics and achieve greater performance by increasing the accepted operating temperatures; TBCs can also be used in many applications, such as combustion engines (internal burners and gas turbines) [14,15]. TBCs are a practical approach to eliminate the unburned emissions of hydrocarbons resulting from incomplete combustion. Moreover, a TBC improves the piston surface’s thermal properties by decreasing the thermal conductivity and increasing the unburned charge oxidation [16]. A progressively important technique for CMC coatings is the thermal spraying barrier. ZrB_2_-SiC is a composition capable of rising the maximum service temperature by deposition on a graphite substrate. Moreover, the SiC-based UHTC-sprayed plasma coatings have proven to be of interest to SiC/SiC and C/SiC composites [17,18]. Indeed, it was once stated that the oxidation and SiC decomposition depends on the UHTC coating content of SiC [19]. Although ZrB_2_-SiC could lead to low levels of SiC, SiC decomposition during spraying has not been observed [20,21,22,23]. This is due to the formation of liquid phases, which is lower than the decomposition of SiC. The eutectic phase at 2207 °C [24] is immediate, as indicated by the ZrB_2_-SiC pseudo-binary phase graphic (Figure 1) Several studies have used the composition either to analyze the phase diagram in the vicinity of ZrB_2_ or to study the eutectic phase (near the SiC axis) phase diagram in opposite ways, as shown by the dotted line [19,24].

A SiC-reinforced ZrB_2_-based UHTC was built by Zhang et al. [25]. The defensive capabilities of these models have been demonstrated with a heat-pressing procedure at high temperatures (>2300 °C). The stability and oxidation strength of UHTCs depend mostly on simulated re-entry conditions in the atmosphere [25,26]. However, at higher temperatures (above 2300 °C), the oxidation and removal of the material do not demonstrate ample resistance to the aerothermal heat load that is being applied. In addition to oxidation during functional operations, mechanical loads should be considered. ZrO_2_ can provide the configurable stability of the substance, here with the oxide frame not having to be removed by the flow of the gas. The temperature limits of UHTC are subject to the relaxation and degradation of the ZrO_2_-based oxide scale, which is also regulated by SiC material because nearly all SiO_2_ material is compared with the melting point of the ZrO_2_-based oxide scale, where SiC content is controlled. When the shear forces go beyond the strength of the shaped oxide structure during the ablation process, the oxides peel off from the specimen, causing increased oxidation and configuration degradation and impacting the vehicles’ performance and protection.

In the current paper, we propose the creation of a microstructure coating that will advance plasma spraying technology in CMC applications and protect against high temperature oxidation. This method has the benefit of being able to choose from a large material for the spraying and substrate; however, the deposition of thick coatings is also difficult. Using well-established coatings, the use of UHTC compositions can be carried out to protect materials at high temperatures (e.g., higher SiC content or decreased oxidation resistance compared with the low SiC contents of UHTC) using various surface treatment conditions (torch vs. furnace). The formation of an adherent, multiphase and protecting oxide scale on the exposed surface is been analyzed, along with the morphology and oxidation behavior. For high SiC content dependent UHTC coatings, the oxidation properties are rarely observed. Graphite substrates promise to be light and retain mechanical properties at high temperature, but when they expose to oxygen containing atmosphere, they immediately burn. What made this outcome more exceptional was that the oxidation property enhancements were achieved.

## 2. Experimental

### 2.1. Substrate Surface Treatment and Deposition Process

Graphites were used as a substrate-reinforcing material for ceramic with two different thicknesses due to their different resistance to thermal shock and oxidation. Graphites with thicknesses of 3 mm and 1 mm were purchased from Mersen (Istanbul, Turkey), carrying the trade names Cerbertie^®^ CM80 and C50, respectively. To improve coating/substrate matching, both in terms of substrate deformation and coating adhesion, different scans during the high-pressure plasma spray (HPPS) process and different surface treatments before the deposition process were tested. The number of total HPPS scans was 20 and 10, respectively.

The ZrB_2_ and SiC commercial powders were supplied by Centro Sviluppo Materiali (CSM, Rome, Italy) and had an average size of 2 μm and 700–800 nm, respectively. ZrB_2_ and SiC were mixed to obtain the powder compositions of ZrB_2_ 50 vol.%-SiC 50 vol.% and of ZrB_2_ 70 vol.%-SiC 30 vol.%. The mixture was agglomerated using a spray-drying process, producing a spherical grain powder with an average grain size of 60 μm, which is appropriate for thermal spray deposition. Scanning electron microscopy (SEM) (JEOL JSM-IT300, Tokyo, Japan) was used to examine the agglomerated particle size, while the phase composition was analyzed by energy dispersive X-ray spectroscopy (EDS) (Oxford instrument, Oxford, UK).

Table 1 lists all the HPPS deposition processes performed. The two different graphite substrates are reported as substrate A (3 mm thickness) and substrate B (1 mm thickness). Table 1 indicates the identification number of the deposition process (ID), type of SiC-ZrB_2_ mixture, total number of scans performed, and average thickness of the deposited coating.

Processes A and B were performed to test the HPPS process parameters and to verify good matching between the coating and different graphite substrates. In particular, because of the different substrate thicknesses, different scans were performed in the HPPS deposition treatments to reduce the deformation of the graphite substrates caused by the coating. The number of scans tested had been 20 and 10 for specimens A and B, respectively, to pursue an average coating thickness above 100 microns. A surface pretreatment before the deposition process was tested to improve the coating-substrate adhesion. Both the untreated and paper-treated surfaces by hand abrasion (grain size P120) were tested for specimens A and B. Specimen A did not require any kind of surface treatment because it had the proper roughness. The best coating–substrate matching was observed for substrate A, both in terms of substrate deformation and coating adhesion.

The SiC-ZrB_2_ coatings were carried out using Plasma-Technik AG (Wohlen, Switzerland) to control the deposition atmosphere and pressure in the chamber during the coating process. The graphite substrate was placed on a metal frame to ensure there was no movement during the coating process. The frame with the substrate was placed on the plasma chamber under an argon atmosphere. The samples were then exposed to elevated enthalpy plasma flows using the arc-jet facility equipped with a 40 kW plasma torch operated in an inert gas (He, N2, Ar, and their mixtures) at mass flow rates of up to 5 g/s. The samples were located 11.0 cm away from the exit torch. The different number of torch scans was performed to reduce the deformation of the graphite substrates; this resulted in the coating. The coating parameters are summarized in Table 2. The parameters were chosen for the experiments with different substrate thicknesses, initial powder compositions and number of scans in order to prepare the design of these coated materials with customized property profiles.

### 2.2. Coating Characterizations

The microstructure and morphology of the samples were examined using a transmission electron microscope (TEM) (JEOL JEM-2100Plus) (JEOL Ltd, Tokyo, Japan) and an SEM (JSM-IT300, Tokyo, Japan), respectively. The JEOL JEM-2100Plus TEM was fitted with an Oxford energy-dispersive X-ray spectrometer (Oxford instrument, Oxford, Oxfordshire, UK). EDS for the composition study was performed using scanning transmission electron microscopy (STEM) (JEOL Ltd, Tokyo, Japan) mode.

**Figure 2:**
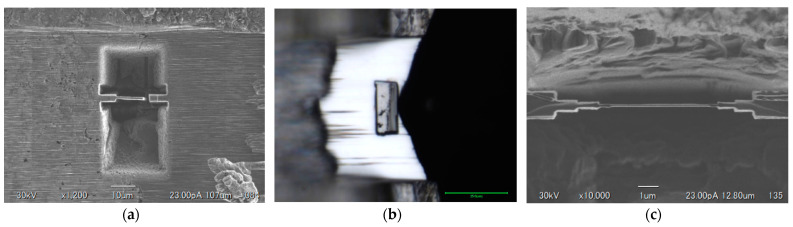
Images illustrating bulk pickup method (**a**) side cut (**b**) after pickup and (**c**) thinning to 90 nm.

Before the sample was examined on TEM, a multistep process using a JIB-4000PLUS (Jeol) focused ion beam (FIB) system was used to prepare a thin-film of the coated specimens as shown in Figure 2. This process has the advantage of examining the ZrB_2_-SiC coated thin-film in TEM. The images illustrate that the block samples were mechanically cut with FIB, sample block pickup, and thinning process.

The X-ray diffraction (XRD) of ceramic samples was obtained by using a Jeol X-ray diffractometer (model) (JEOL JDX-8030, Tokyo, Japan) using Cu-K_α_ radiation, which generated a voltage of 40 kV and current of 40 mA (λ = 1.54A°). The diffraction angle of 2θ was scanned from 5 to 80° at a scanning rate of 2°/min and a step size of 0.2°. An XRD analysis of the ceramic samples was done in plate form. The average crystallite size of the ZrB_2_ and SiC particles was calculated using the Scherrer equation.

The bulk density and theoretical density were evaluated using the Archimedes method (water as an immersing medium) for the mixture. The relative density was calculated by dividing the bulk density by the theoretical density (densities of 6.09 g/cm^3^ for ZrB_2_ and 3.21 g/cm^3^ for SiC were used for the theoretical densities’ calculation).

A laser flash analysis using Netzsch LFA 467 HyperFlash^®^ (Erich NETZSCH GmbH & Co. Holding KG, Selb, Germany) was used to assess thermal diffusivity. The tests were performed in an argon atmosphere of up to 450 °C. The samples were cut using an Electronica EcoCut Machine (Electronica, kolkata, West Bengal, India) with a 12 mm diameter; otherwise, a Teenking (TK-TRUMP50-G3020) Waterjet Cutting Machine (Teenking CNC Machinery Co., Ltd., Foshan, Shunde district, China) was used to cut the samples for the other analyses.

A thermal analysis (furnace test) was performed on 35 × 35 mm samples in a high-temperature furnace in air at different temperatures (at 800 °C, 1000 °C, and 1200 °C) for 30 min. The original dimensions of the coated samples were 70 × 70 mm, and they were cut using a water jet (Teen King Water jet tk-trump50—G3020). For analysis preparation, all samples were washed with acetone and dried in a vacuum furnace for 48 h. The heating rate was 10 °C/min, and the samples were left to cool to room temperature after treatment.

**Figure 3:**
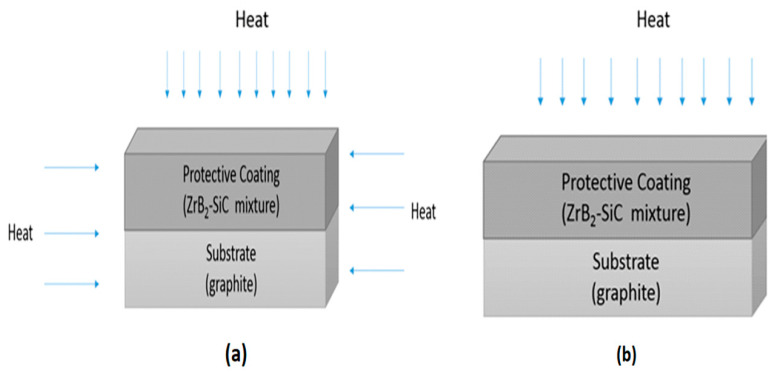
Schematics illustrating thermal analysis (**a**) furnace test and (**b**) torch test.

Besides furnace thermal analysis, the oxidation resistance properties of the prepared materials were investigated by the torch test because of the graphite oxidation (from sides) by air at temperatures higher than 450 °C [19], as shown in Figure 3. A torch thermal analysis was performed on 35 × 35 mm samples at 800 °C, 1000 °C, and 1200 °C for 5 min, and a mixture of methane-oxygen was used for this analysis. For the analysis preparation, all samples were washed with acetone and dried in a vacuum oven for 48 h.

## 3. Results and Discussion

### 3.1. Structure of SiC-ZrB_2_ Starting Powder and Coatings

**Figure 4:**
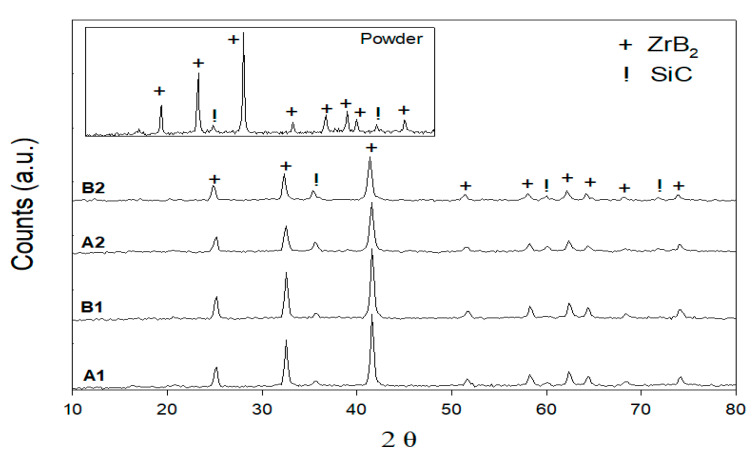
XRD patterns of SiC-ZrB_2_ starting powder and coatings.

The starting powder and coating samples were all performed with XRD. The XRD patterns are shown in Figure 4. The XRD patterns can be entirely indexed to ZrB_2_ and SiC, that is, only the initial phases are included in the coating. Additional phases were not observed, such as for SiO_2_, ZrO_2_, B_2_O_3_, or ZrSiO_4_. The absence of oxide stages in the coating is because of the parameters of spraying used (in inert atmosphere deposition, H_2_ as plasma gas). However, the XRD patterns suggest that the preservation of ZrB_2_ and SiC phases in the deposited coatings and the chemical elements analysis features a quantity of silicon in good agreement with the starting powders as shown in Table 3. Earlier works [18,19,20,21,22,23] showed that if the SiC content is lower than the ZrB_2_ content, ZrB_2_-SiC coatings could be produced. SiC decomposition, as seen in the pseudo-binary phase diagram for ZrB_2_-SiC (Figure 1), does not occur during spraying because of the fluid phase formation at a temperature below SiC decomposition, when there is a 2207 °C eutectic process [24]. The signatures of second and third SiC peaks in the ZrB_2_-SiC B1 mixture (70%-30%) are not detected using XRD between 2θ = 60° to 80° and the phase composition periodicity can only be detected at lower angles.

**Figure 5:**
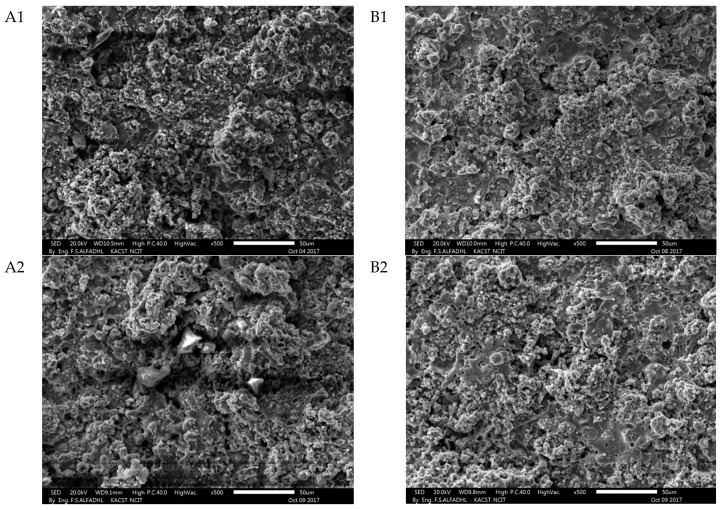
Secondary electron SEM images of the surface of the SiC-ZrB_2_ for A1, B1, A2 and B2 coatings taken at 20 kV electron acceleration voltage at 500× (scale bar = 50 μm) low magnifications.

**Figure 6:**
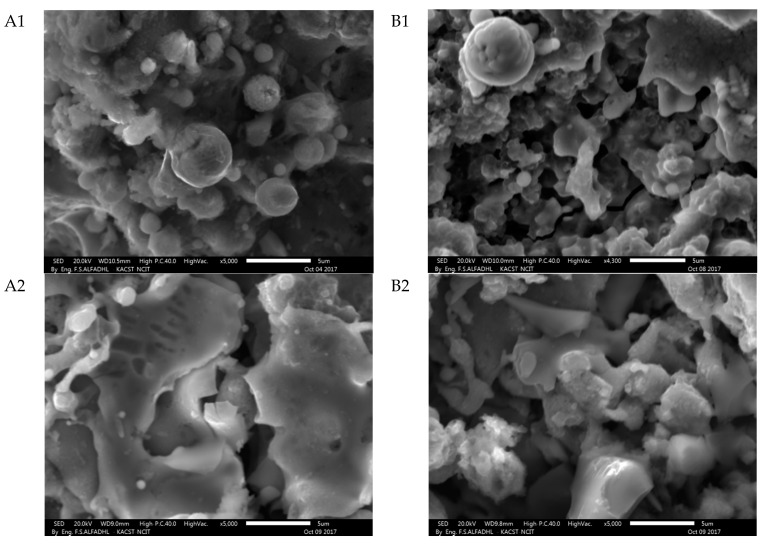
Secondary electron SEM images of the surface of the SiC-ZrB_2_ for A1, B1, A2 and B2 coatings taken at 20 kV electron acceleration voltage at 5000× (scale bar = 5 μm) high magnifications.

This coating composition is expected to proceed with the above-mentioned liquid phase formation process below the SiC decomposition temperature. Figure 5 and Figure 6 show the surface of the A1, B1, A2, and B2 SiC-ZrB_2_ coating observed by SEM for low magnifications: it appears to be compact and uniform (Figure 5), and no unmelted particles were observed (details of ZrB_2_-SiC mixture particles and EDS analysis images shown in the Supplementary Materials Figures S1–S15). The observed variance of gray colors indicates the presence of various Zr and Si compounds. EDS analyses verified that the SiC particles are composed of dark gray regions, ZrB_2_ particles are made of white zones, and Zr- and Si-containing gray-intensity middle zones are present. The theory of a fluid phase formation based on the phase diagram seems to be supported by metallographic research. The coating tends to be shaped in high sizes by splats of various morphologies at high magnifications (Figure 6). This can be clarified if the substrate is affected by certain particles in a part-molten state, that is, within the solidus–liquid range at a temperature; other particles have melted entirely, that is, above the temperature of the liquid. These two circumstances correspond to points A and B of Figure 1, respectively. This can be attributed to: (a) different sizes of the particles, (b) different compositions (flowing around the nominal), and (c) different thermal backgrounds during spraying.

**Figure 7:**
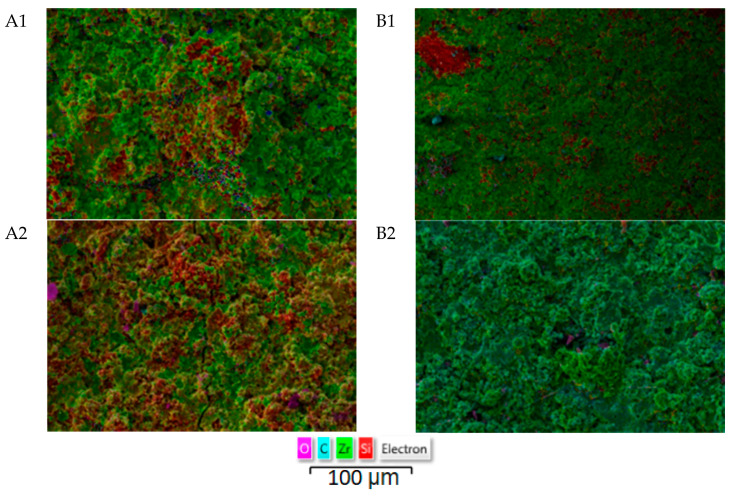
EDS of SiC-ZrB_2_ for A1, B1, A2 and B2 coatings.

A fast cooling rate, which is common for spraying with low miscibility of the ZrB_2_ and SiC phases and that reduces the kinetic crystallization, causes a strong impact on the splat morphology. The EDS surface coating analyses recorded in Figure 7 reveal that the surface is comprised of a Zr-enriched matrix of SiC particles (average size < 1 μm). Therefore, a particle in a partially melted state might produce this splat (point A of Figure 1).

**Figure 8:**
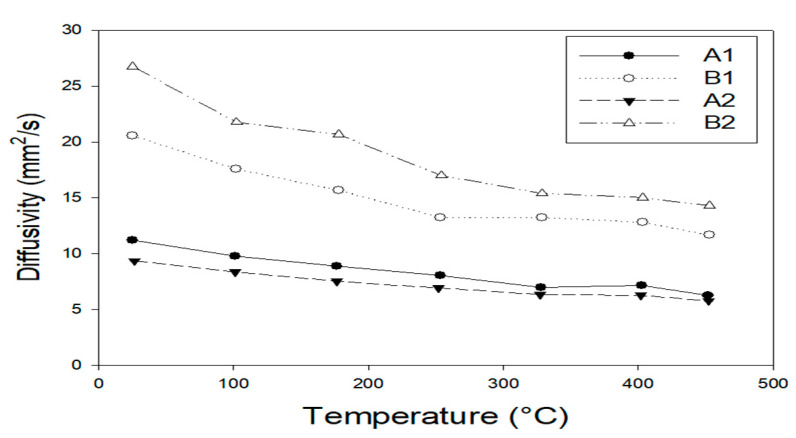
Diffusivity as a function of temperature for A1, B1, A2, and B2.

For all samples in Figure 8, the thermal diffusiveness versus temperature, curves are shown. The diffusiveness of both the SiC-ZrB_2_ mixtures increases with a greater content of SiC and reduces the layer thickness of the deposited coating, with room temperature values ranging from 21.0 cm^2^/s for the B2 layer to 27.5 cm^2^/s for the B1 layer. In addition, the coating/substrate matching of the A1 and A2 layers suggests slightly but consistently lower diffusivity values at high temperatures.

### 3.2. Oxidation Behavior

**Figure 9:**
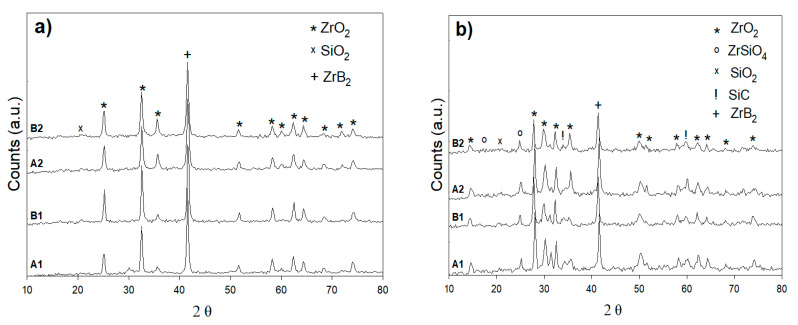
XRD patterns of the surface oxidation of A1, B1, A2, and B2 of (**a**) torch treatment for 5 min at 800 °C and (**b**) furnace treatment for 30 min at 800 °C.

**Figure 10:**
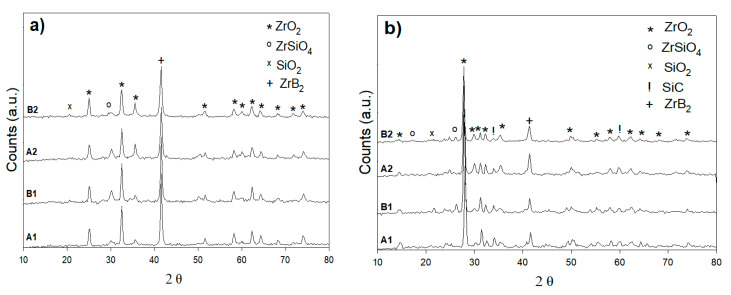
XRD patterns of the surface oxidation of A1, B1, A2, and B2 of (**a**) torch treatment for 5 min at 1000 °C and (**b**) furnace treatment for 30 min at 1000 °C.

**Figure 11:**
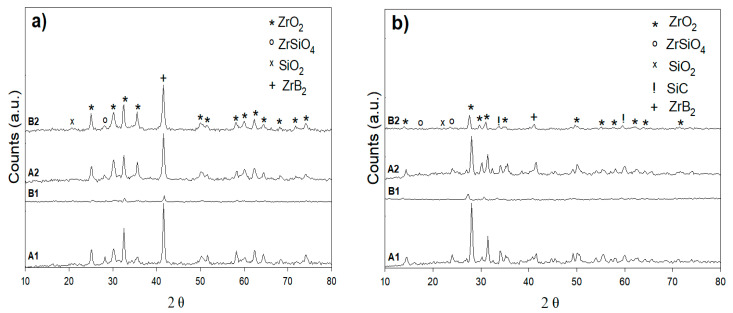
XRD patterns of the surface oxidation of A1, B1, A2 and B2 of (**a**) torch treatment for 5 min at 1200 °C and (**b**) furnace treatment for 30 min at 1200 °C.

A gas torch test was given for relative exposure times (5 min) and a furnace treatment test for 30 min after long-term oxidation up to 1200 °C in preparation for UHTC compositions of SiC-ZrB_2_. The torch test was conducted for XRD patterns A1, B2, A2, and B2, which are comparable with those XRD patterns of the composites tested in the furnace at 800 °C (Figure 9a,b). The XRD patterns of both composition measures is shown in Figure 9a. Here, 70% SiC–30% ZrB_2_ and 50% SiC–50% ZrB_2_ are very different from those in Figure 9b: the ZrB_2_ peaks also were present after oxidation, but silica was the only extra surface step. Oxygen, silicone, and boron were primarily the exterior glass layer of the furnace treatment; in addition, some traces of zirconium existed. A sample with a 30% SiC–70% ZrB_2_ had a content of Si; thus, O was higher than in the 50% SiC deposited coatings and displayed a more Si-rich glass-like phase with a rising SiC level. The appearance of a diffraction peak corresponding to ZrO_2_ superimposes a broad signal that was characteristic of the presence of an amorphous phase such as a glassy one. The XRD spectra obtained from the oxide scales formed during isothermal exposure at 1000 °C and 1200 °C (torch compared to the furnace test) are shown in Figure 10a,b and Figure 11a,b respectively. The results showed that the principal phase in an oxide scale was monoclinic ZrO_2_, while tetragonal zirconia was not found in the torch setting for the samples oxidized at 800 °C. Tetragonal zirconia may be able to form during oxidation but can also be transformed during cooling into monoclinic zirconia [14,27]. In the sample oxidized at 1000 °C and 1200 °C, some traces of zircon (ZrSiO_4_) were detected. While the formation of stable zircon may be predicated based on a ZrO_2_-B_2_O_3_-SiO_2_ system ternary isothermal segment at 1000 °C, this compound is frequently not recorded in high-temperature oxidized ZrB_2_-SiC composites (>1500 °C) [27,28]. It is worth noting that in the B1 XRD pattern at 1200 °C, a bump due to the amorphous SiO_2_ can be observed while in the XRD pattern at 1000 °C crystalline SiO_2_ (cristobalite) exists; this is the outcome from a continuous devitrification of the amorphous silica film [18].

**Figure 12:**
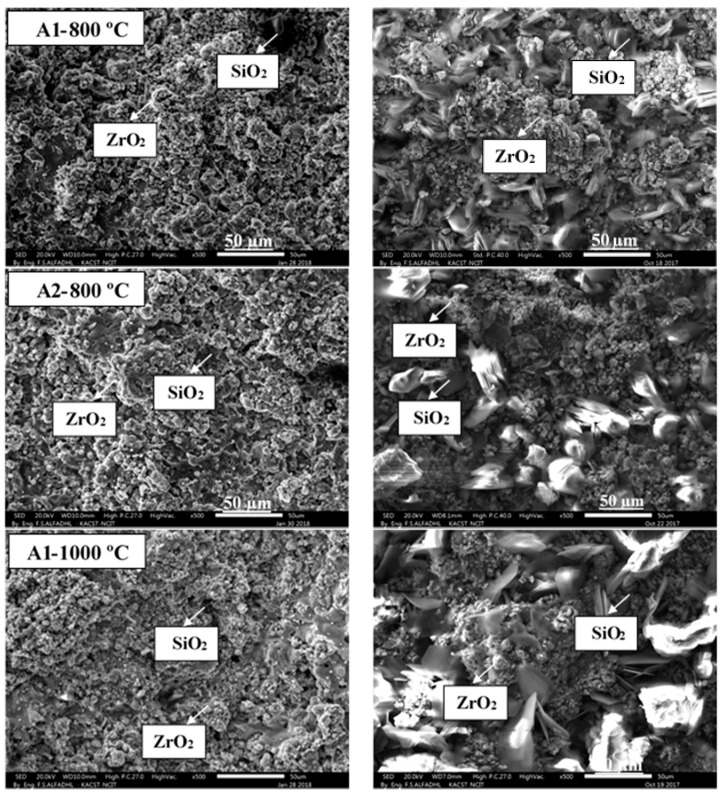

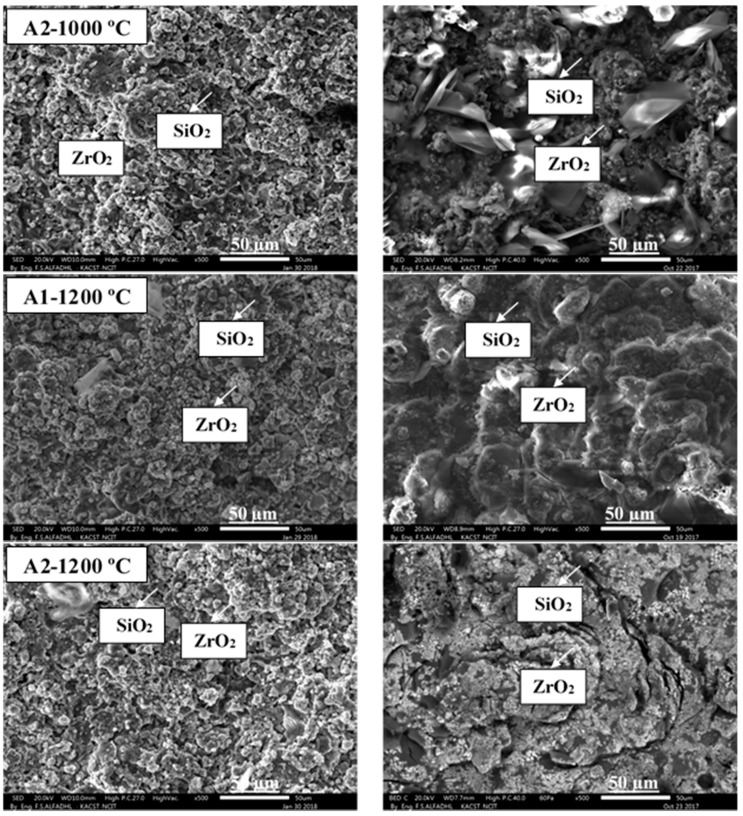
Secondary electron SEM microstructure of the A1 and A2 with different surface treatment conditions torch (**left**) and furnace (**right**) at 800 °C, 1000 °C and 1200 °C coatings taken at 20 kV electron acceleration voltage at 500× (scale bar = 50 μm) magnifications.

SEM analyses were conducted on sample surface at 800 °C and 1000 °C for A1 and A2, as defined in Figure 12. The findings of the SEM observation are consistent with the suggested oxidation mechanism [29]. The polygonal grains of B_2_O_3_ could be observed on a sample surface exposed to a furnace at 800 °C and 1000 °C; the grains correspond to the formation in the initial stage of the first B_2_O_3_ nucleuses. Conversely, B_2_O_3_ is already formed in the sample exposed at 1200 °C and partly covers the sample surface. The samples can contain unoxidized SiC particles. The A2 sample reveals an oxidized film of pore-like zirconium oxide and silicone oxide islands exposed to a temperature of 1200 °C. Samples exposed to higher temperatures have been documented as being to compact silicon oxide scale, including ZrO_2_ [19]. A comparison of the SEM-surface micrographs of torch-oxidized specimens reveals that the oxide level produced was rougher and grainier than that produced in the furnace. With the extensive formation of bubbles, all these oxide levels appear porous. The surface was often protected by a dense silica layer of glass (800 °C, 1000 °C and 1200 °C) with aggregated zirconia in various sizes and types. This aspect of the surface conforms to the ZrB_2_-SiC bulk composite oxidation mechanisms described in [29]. In fact, a borosilicate oxide (B_2_O_3_-SiO_2_) film forms on the outer surface during high-temperature oxidation of ZrB_2_-SiC. Here, boria is preferentially evaporated from the fluid borosilicate because of the high vapor pressure of boria at these temperatures relative to silica. The liquid oxide film on the outside then becomes a primarily viscous SiO_2_-rich fluid that covers and fills the pores of the porous oxide dimension. Gas product coalescence (i.e., CO) within the external-forming glass most likely contributes to the creation of large bubbles. The key difference caused by exposure temperatures is that the oxide scale has risen and that the zirconia particles decreased simultaneously on the surface, with cracks and discontinuities being found.

**Figure 13:**
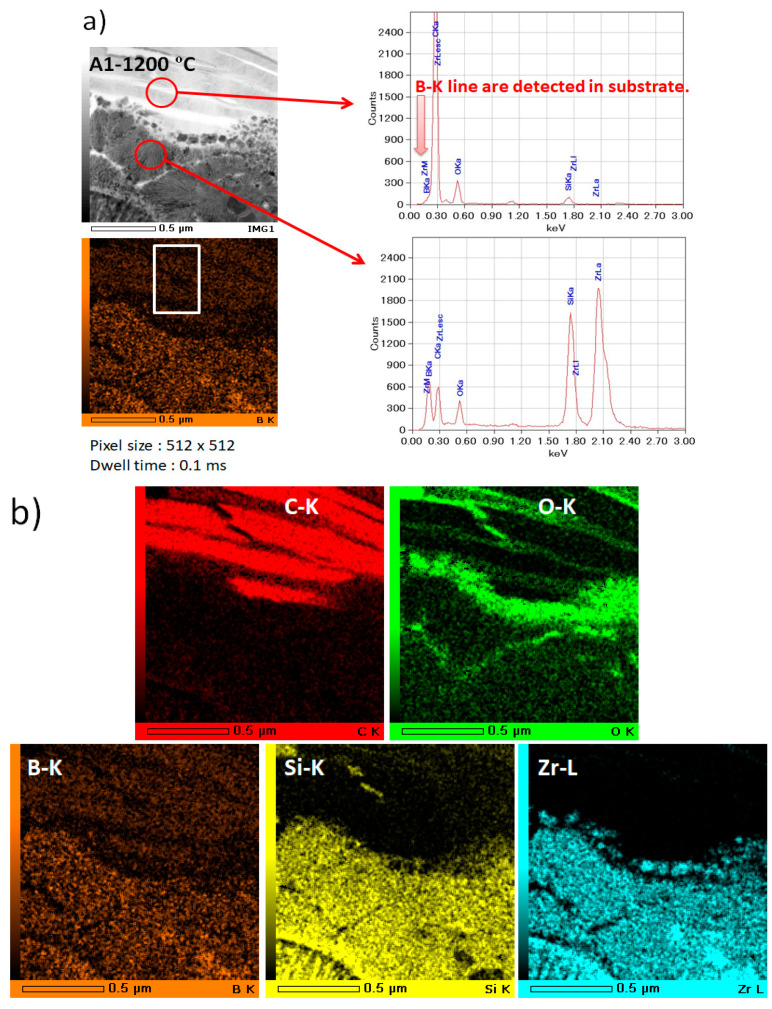
EDS/STEM analysis of coated sample A1 (**a**): STEM image with arrows indicating the EDX line scan position with Si Kα signal and B Kα signal of the particles; (**b**): the distributions of elements in 70%SiC-30%ZrB_2_ at 1200 ºC.

**Figure 14:**
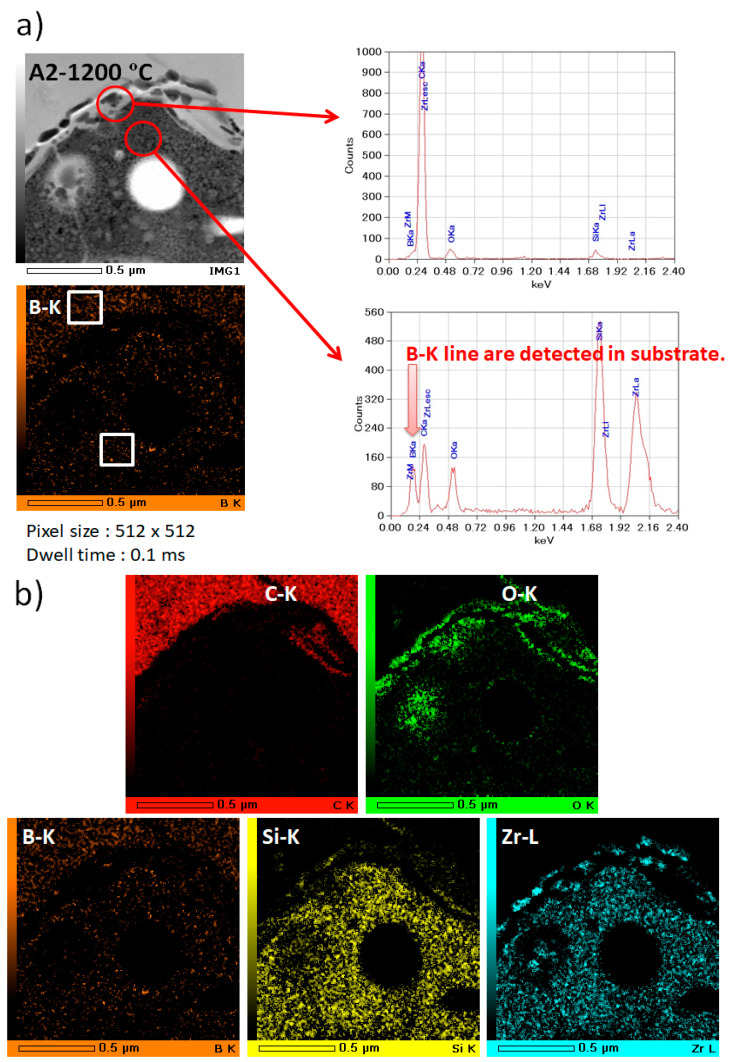
EDS/STEM analysis of coated sample A2 (**a**): STEM image with an arrows indicating the EDX line scan position with Si Kα signal and B Kα signal of the particles; (**b**): the distributions of elements in 70%SiC-30%ZrB_2_ at 1200 ºC.

The phase and microstructure of the sample A1 and A2 at 1200 °C using a furnace were studied by TEM. Figure 13 and Figure 14 display ZrB_2_ grain chemical mapping of the SiC matrix and substrate in the TEM picture and 2D atomic resolution. ZrB_2_ was directly oxygen-prone in contact with depleted SiC grains, oxidated (Equation (1)), and formed an oxide layer consisting mainly of ZrO_2_.

2ZrB_2_ + 5O_2_ ↔ 2ZrO_2_ (solid) + 2B_2_O_3_ (liquid) (1)

The TEM results are well in line with the XRD data shown in Figure 11b. The bright field (BF) picture reveals that the grain size of A2 is less than A1. SiC particles are also distributed on the substrate’s grain boundaries. Figure 13a and Figure 14a show that Zr, Si, O, B, and C are composed of different positions along the red arrows in the interface region. The analysis of those figures indicates that as indicated by the EDS maps (Figure 13b and Figure 14b), the oxidizing scale consists of a continuous SiO_2_-rich layer containing different quantities of particulate zirconia. To restrict the internal diffusion of oxygen into the inner bulk and, thus, increase resistance to oxidation, the formation of an external glass layer based on silica was found to be extremely successful. However, oxide layers of boron were found, presumably because of the formation of B_2_O_3_. This item should be viewed as ZrB_2_ particles under oxidized layers when evaporating above 1100 °C [19,20]. The ZrB_2_ synthesis process also provides possible explanations for the existence of this boron-containing step. Indeed, ZrB_2_ is commonly produced from boron oxide, zirconium oxide, and carbon at temperatures above 2400 °C in an arc furnace by means of a process boron-carbothermal [30]. The following reaction (Equation (2)) occurs simultaneously:

B_2_O_3_ + ZrO_2_ + 5 C → ZrB_2_ + 5 CO (2)

In addition, Table 4 shows the relative density. Each sample had similar results because of samples that had the same components. This is predicted. In addition, the thermal treatment of oxidized samples, particularly in the case of B2, has a somewhat lower density effect. The difficulty of processing and manufacturing of the carbide based UHTMs to have enough density was previously reported by Hilmas et al. [4] who studied the oxidation conditions. The transition metal carbides create a single phase (porous metal dioxide scales) that degrade the material. In contrast, transition metal boride shows very high oxidation resistance compared with the carbides transition metals as a result of forming oxidation resistance materials (B_2_O_3_ (boria)) that are formed at 1200 °C. B_2_O_3_ provides oxidation resistance by filling the porosity in the fine-grained oxide.

## 4. Conclusions

To test ultra-high-temperature applications, a series of CMCs with various SiC-ZrB_2_ ratios was successfully deposited in a graphite substrate (1 mm and 3 mm thick). The samples were rendered with plasma spraying to improve the coating of the microstructure and prevent the oxidation of the materials. Patterns in the XRD confirmed the preservation of ZrB_2_ and SiC phases in the deposited coatings, and a quantity of silicon consistent with the starting powders was emphasized in the chemical analysis. Coatings with a low porosity content were found to be lightweight and homogeneous. However, the low porosity of CMC samples reveals a lower specific surface area to react with the oxygen, decreasing this approach the quantity of mass gain. As SiC decreased, the CMC samples’ porosity increased, and the mass gain increased accordingly:

ZrB_2_ (s) + 5/2 O_2_ (g) → ZrO_2_ (s) + B_2_O_3_ (s,l)

The substrate with 3 mm thickness brought out the best properties of CMC. Moreover, the increasing SiC content in the composites impart additional benefits in promoting a decrease in ZrB_2_ grain size for the two set of composites.

Oxidation experiments (torch vs. furnace) were conducted in temperatures up to 1200 °C: the material investigated demonstrated its strong protection potential against the CMC hot structures with high-temperature oxidation. After thermal treatment in a furnace, the oxide scales formed on the ZrB_2_-SiC composites were more complex, consisting of the following three sub-layers:

- The outer glass layer consisted primarily of a carbon, including borosilicate glass.
- The intermediate layer was made up of ZrO_2_ plus borosilicate crystalline glass.
- An inner pore layer with a SiC depletion came from SiC to Si and CO active oxidation mechanisms.

The ZrB_2_-SiC B1 mixture (70–30%) submitted to both oxidation treatments at 1200 °C are recommended to study strength and stiffness; the mechanical properties of the oxidized material can be enhanced by increasing the ZrB_2_ ratio.

## Figures and Tables

**Table 1:** List of high-pressure plasma spray (HPPS) deposition processes.

ID	Mixture	Number of Scans	Coating Thickness (µm)
A1	ZrB_2_-SiC (70%-30%)	20	250
B1	ZrB_2_-SiC (70%-30%)	10	130
A2	ZrB_2_-SiC (50%-50%)	20	240
B2	ZrB_2_-SiC (50%-50%)	10	120

**Table 2:** Coating parameters.

Parameter	Value
Pressure (Ar gas)	1200 mbr
Spray distance	110 mm
Plasma gas (Ar)	500 slpm
Plasma gas (H_2_)	150 slpm
Plasma power input	40 kW

**Table 3:** Chemical elements analysis determined from energy dispersive X-ray (EDS).

Chemical Composition	Zr (wt.%)	Si (wt.%)
Starting composition (50%-50%)	30.73	26.18
ZrB_2_-SiC powder	33.90	25.20
ZrB_2_-SiC coating	33.49	26.31

**Table 4:** Coating densities.

Sample	Temperature (°C)	Density g/cm^3^
A1	Room Temperature (RT)	2.02
B1	RT	2.15
A2	RT	2.01
B2	RT	2.07
A1	1200 °C	1.95
B1	1200 °C	2.13
A2	1200 °C	1.97
B2	1200 °C	1.91

## Data Availability

The data presented in this study are available on request from the corresponding author.

## Supplementary Materials

The following are available online at https://www.mdpi.com/1996-1944/14/2/392/s1, Figure S1: SEM image of the coating B2, ZrB_2_-SiC (50%-50%)—metallographic cross section: two halves of the coating cross section are compared (black area = resin), Figure S2: SEM image of the coating B2, ZrB_2_-SiC (50%-50%)—metallographic cross section: details, Figure S3: SEM image of the coating B2, ZrB_2_-SiC (50%-50%)—metallographic cross section: coating thickness, Figure S4: SEM image of the coating B2, ZrB_2_-SiC (50%-50%)—metallographic cross section: coating thickness, Figure S5: SEM image of the coating B2, ZrB_2_-SiC (50%-50%)—metallographic cross section: details, Figure S6: SEM-EDS analysis of the coating B2, ZrB_2_-SiC (50%-50%): area analysis, Figure S7: SEM-EDS analysis of SEM-EDS analysis of the coating B2, ZrB_2_-SiC (50%-50%): point analysis, Figure S8: SEM image of the coating B1, ZrB_2_-SiC (70%-30%)—metallographic cross section: two halves of the coating cross section are compared (black area = resin), Figure S9: SEM image of the coating B1, ZrB_2_-SiC (70%-30%)—metallographic cross section: details, Figure S10: SEM image of the coating B1, ZrB_2_-SiC (70%-30%)—metallographic cross section: coating thickness, Figure S11: SEM image of the coating B1, ZrB_2_-SiC (70%-30%)—metallographic cross section: coating thickness, Figure S12: SEM image of the coating B1, ZrB_2_-SiC (70%-30%)—metallographic cross section: details, Figure S13: SEM image of the coating B1, ZrB_2_-SiC (70%-30%)—metallographic cross section: details, Figure S14: SEM-EDS analysis of the coating B1, ZrB_2_-SiC (70%-30%): area analysis, Figure S15: SEM-EDS analysis of SEM-EDS analysis of the coating B1, ZrB_2_-SiC (70%-30%): point analysis.
