# Supplementary material for: The Oxidation Behavior of ZrB2-SiC Ceramic Composites Fabricated by Plasma Spray Process
Source: Materials (Basel). 2021 Jan 14;14(2):392. doi: 10.3390/ma14020392 (PMC7830389; doi:10.3390/ma14020392)
Supplement: Supplementary file 1 [file materials-14-00392-s001.pdf]

materials

MDPI

Supplementary Materials

The Oxidation Behavior of ZrB_2-SiC Ceramic Composites Fabricated by Plasma Spray Process

Eid M. Alosime, Mohammed S. Alsuhybani and Mohammed S. Almeataq *

King Abdulaziz City for Science and Technology, Riyadh 11442, Saudi Arabia; alosimi@kacst.edu.sa (E.M.A.); sohybani@kacst.edu.sa (M.S.A.)
* Correspondence: mmeataq@kacst.edu.sa

SEM-EDS analysis has been performed to investigate the coating quality and composition, by analyzing both average composition and different phases present in the coating.

Figure S1 shows the SEM images of metallographic coating cross section referred to B2 ZrB_2-SiC (50%-50%) HPPS process. It compares two halves of the coated specimen in order to verify the quality of the coating throughout the cross section. Details of the coating have been investigated in SEM images given in Figures S2–S5. Results of the coating EDS analyses, both as area analysis and point analysis, are given in Figures S6,S7.

Figures S8–S15 show the results of SEM-EDS analysis which have performed for B1 ZrB_2-SiC (70%-30%) process, corresponding to 20 scans process, as it has been considered in the case of B2 ZrB_2-SiC (50%-50%) process.

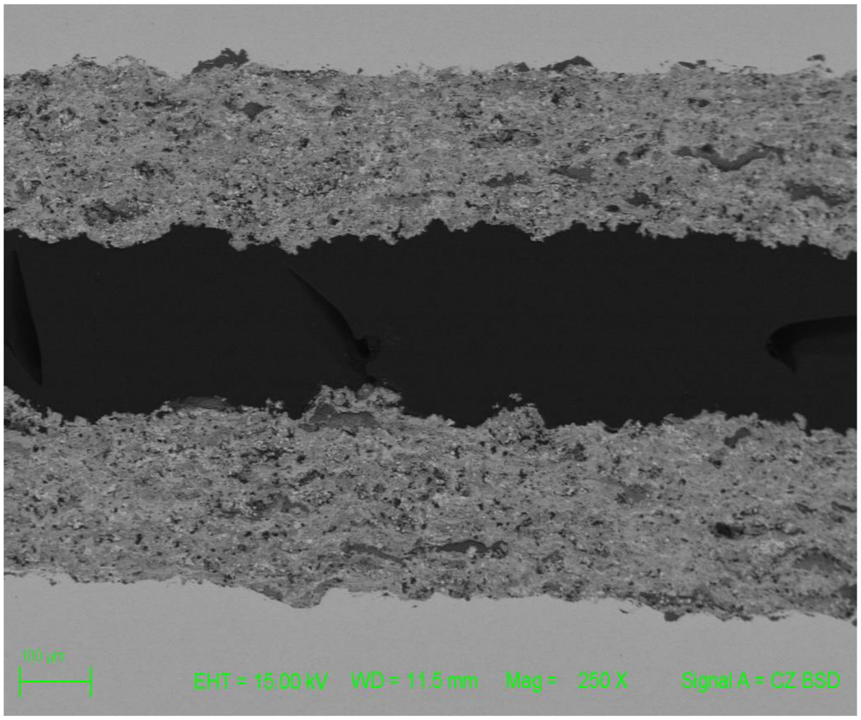

Figure S1. SEM image of the coating B2, ZrB2-SiC (50%-50%)—metallographic cross section: two halves of the coating cross section are compared (black area = resin).

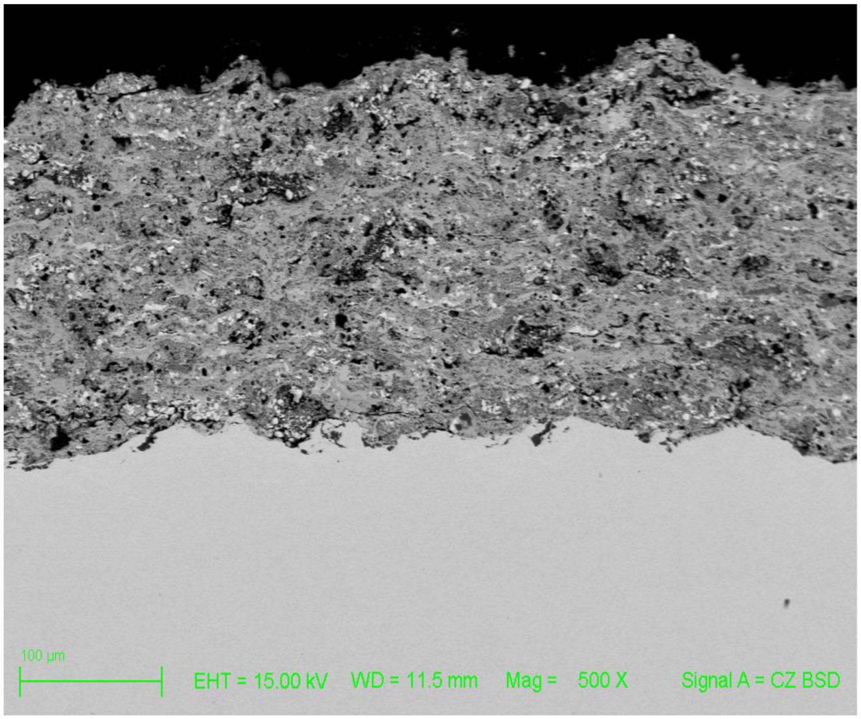

Figure S2. SEM image of the coating B2, ZrB2-SiC (50%-50%)—metallographic cross section: details.

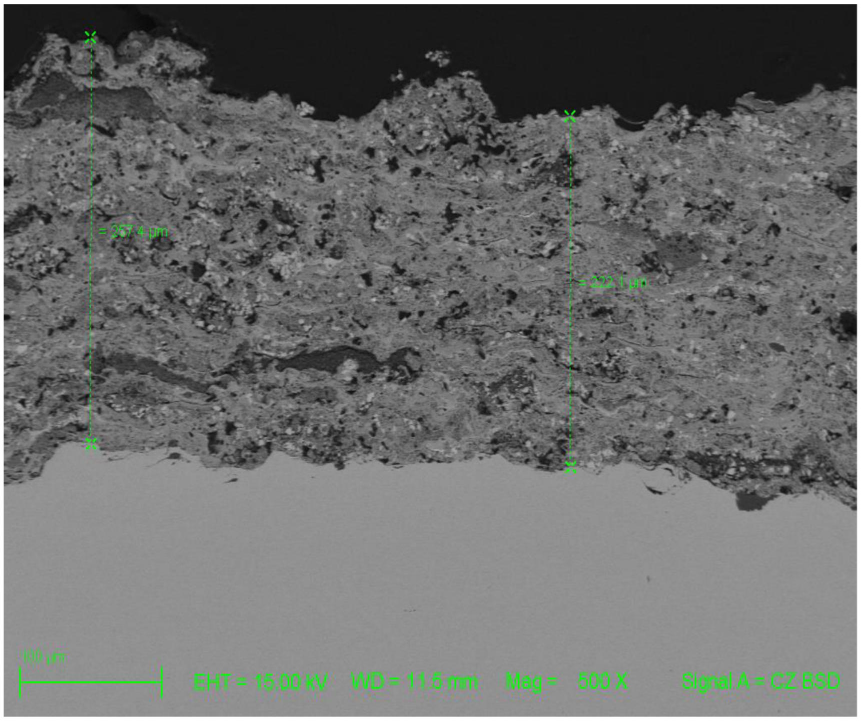

Figure S3. SEM image of the coating B2, ZrB2-SiC (50%-50%)—metallographic cross section: coating thickness.

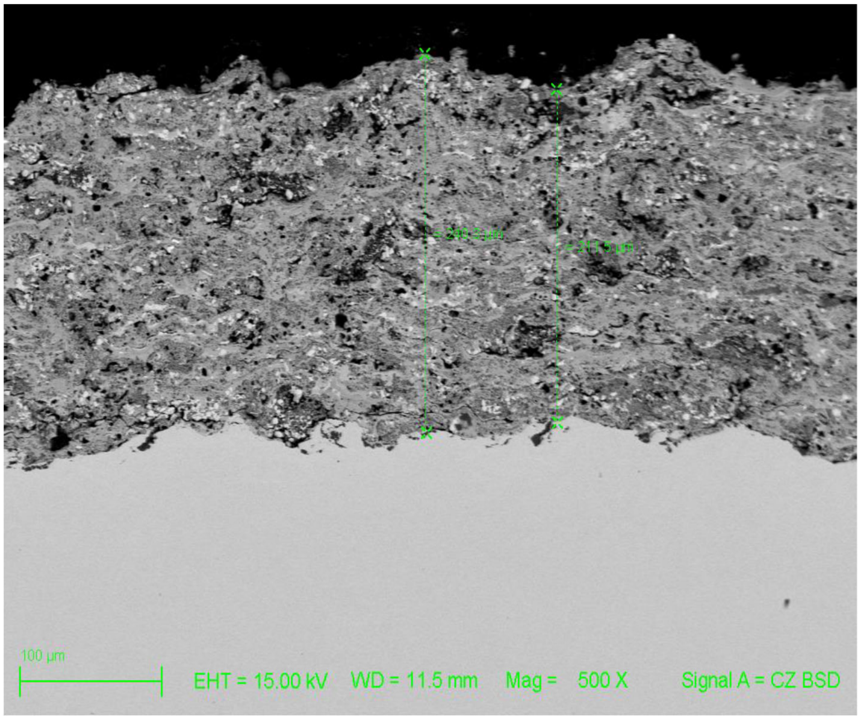

Figure S4. SEM image of the coating B2, ZrB2-SiC (50%-50%)—metallographic cross section: coating thickness.

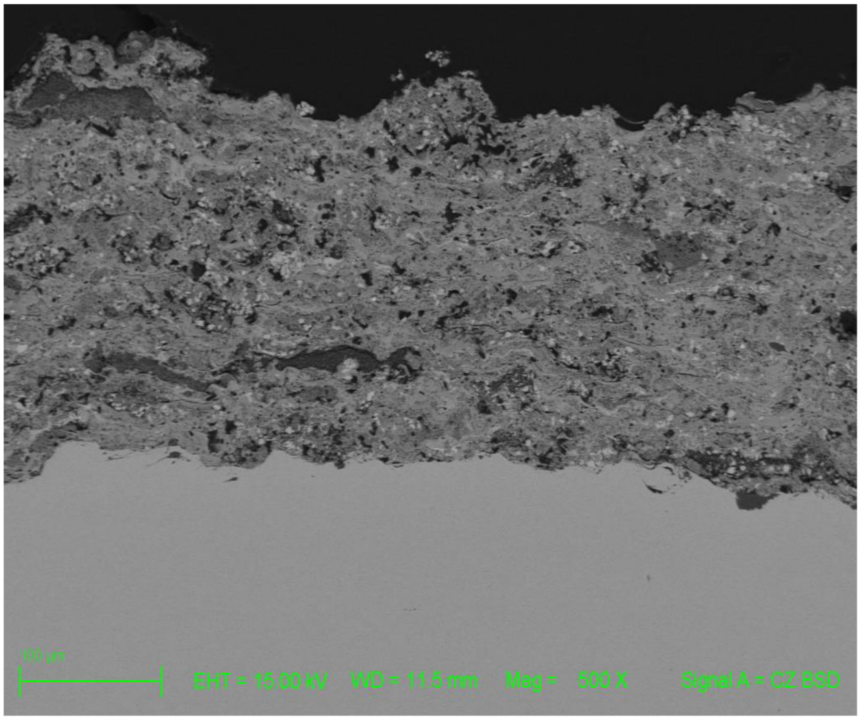

Figure S5. SEM image of the coating B2, ZrB2-SiC (50%-50%)—metallographic cross section: details.

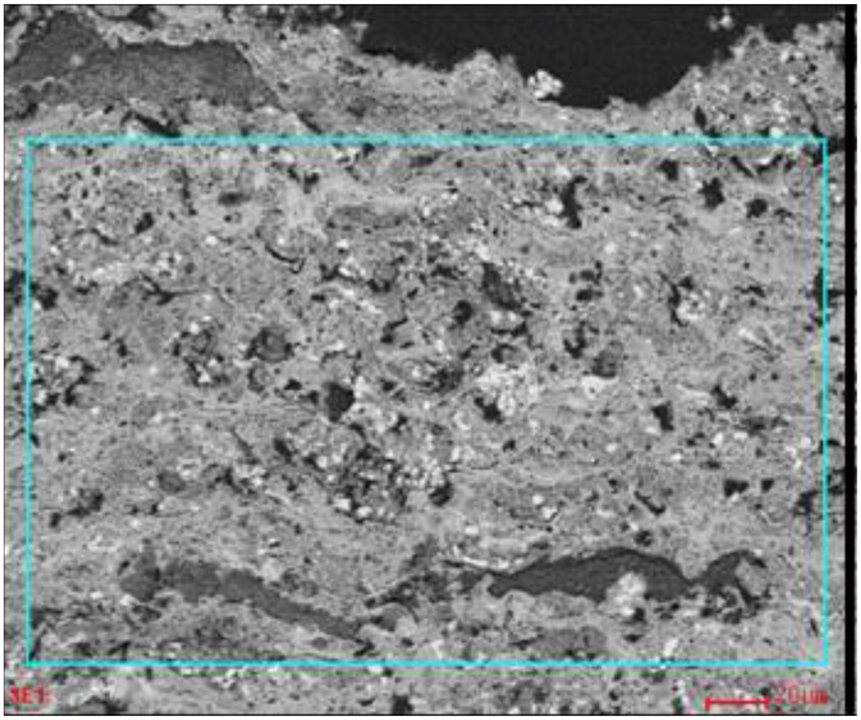

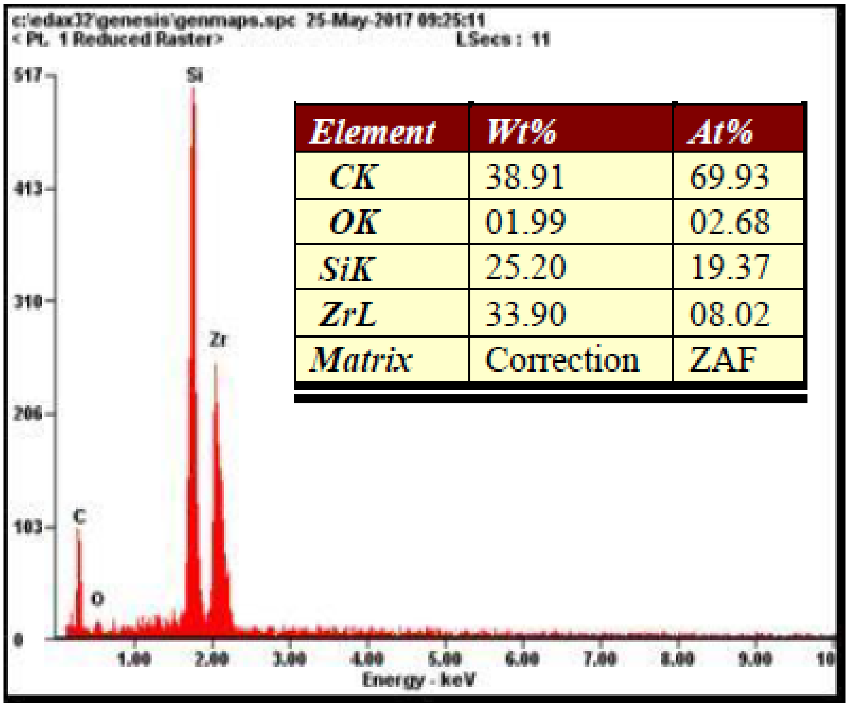

Element	*Wt%*	*At%*
CK	38.91	69.93
OK	01.99	02.68
SiK	25.20	19.37
ZrL	33.90	08.02
Matrix	Correction	ZAF

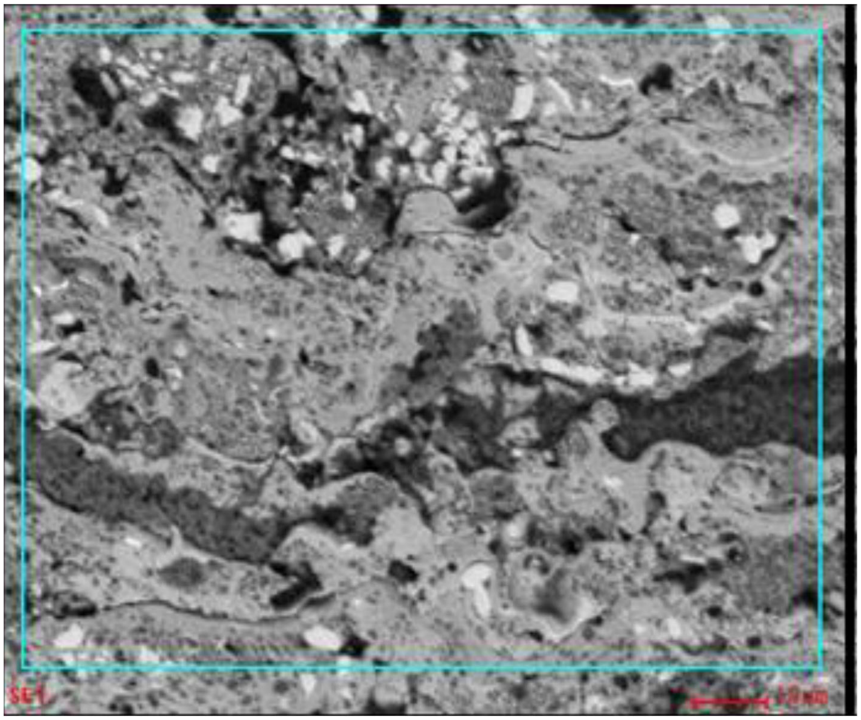

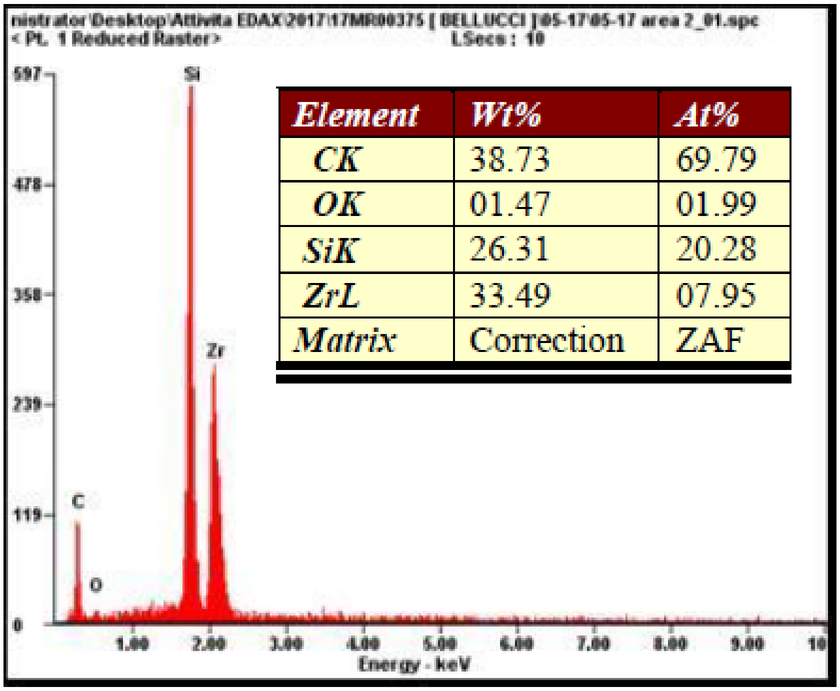

Element	Wt%	At%
CK	38.73	69.79
OK	01.47	01.99
SiK	26.31	20.28
ZrL	33.49	07.95
Matrix	Correction	ZAF

Figure S6. SEM-EDS analysis of the coating B2, ZrB2-SiC (50%-50%): area analysis.

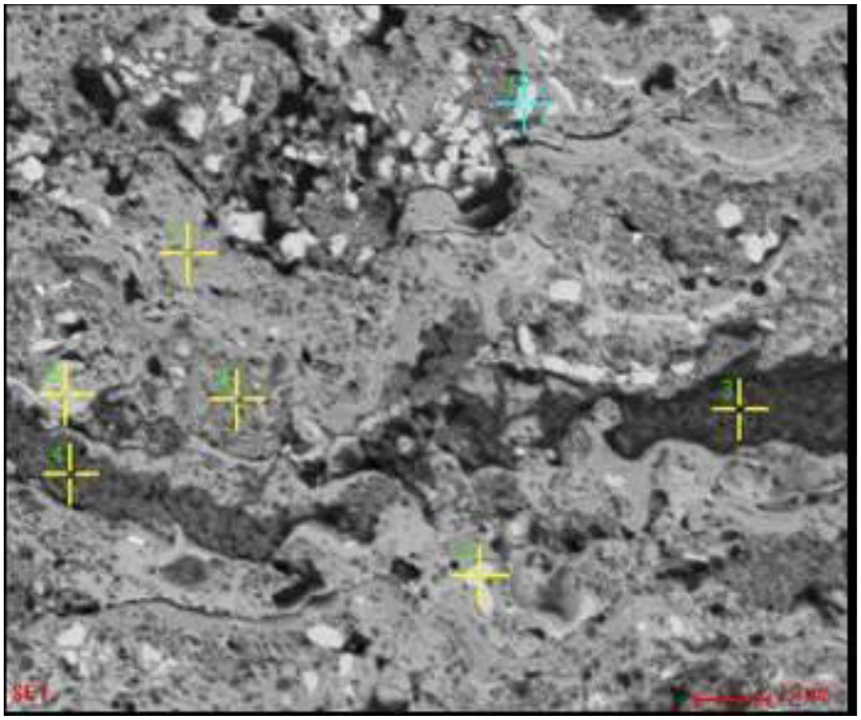

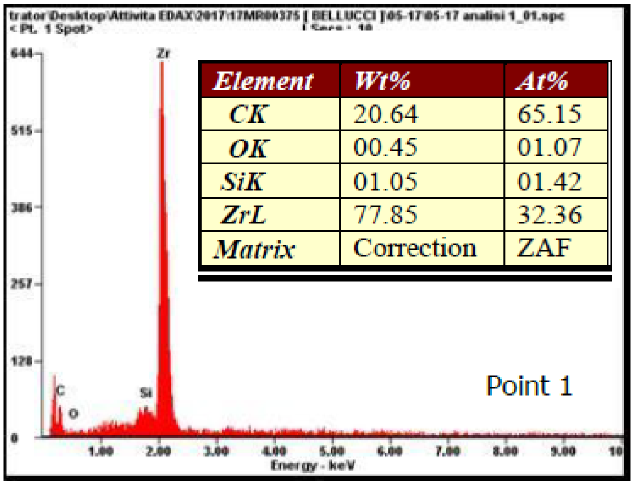

Element	*Wt%*	*At%*
CK	20.64	65.15
OK	00.45	01.07
SiK	01.05	01.42
ZrL	77.85	32.36
Matrix	Correction	ZAF

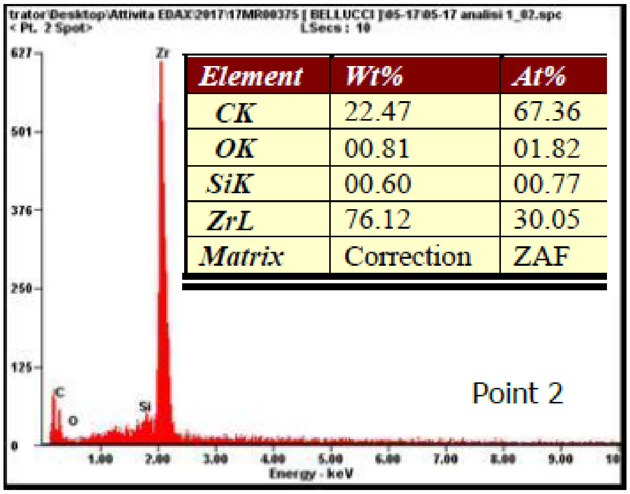

Element	Wt%	At%
CK	22.47	67.36
OK	00.81	01.82
SiK	00.60	00.77
ZrL	76.12	30.05
Matrix	Correction	ZAF

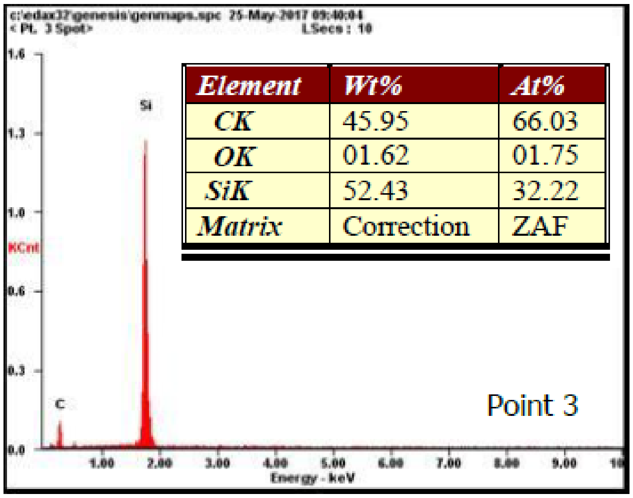

Element	Wt%	At%
CK	45.95	66.03
OK	01.62	01.75
SiK	52.43	32.22
Matrix	Correction	ZAF

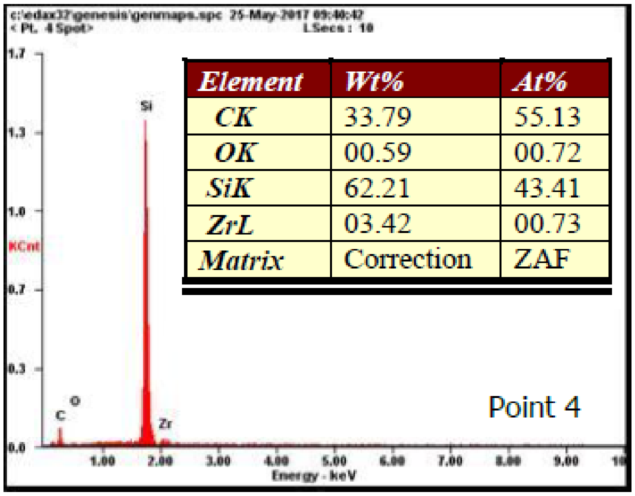

Element	Wt%	At%
CK	33.79	55.13
OK	00.59	00.72
SiK	62.21	43.41
ZrL	03.42	00.73
Matrix	Correction	ZAF

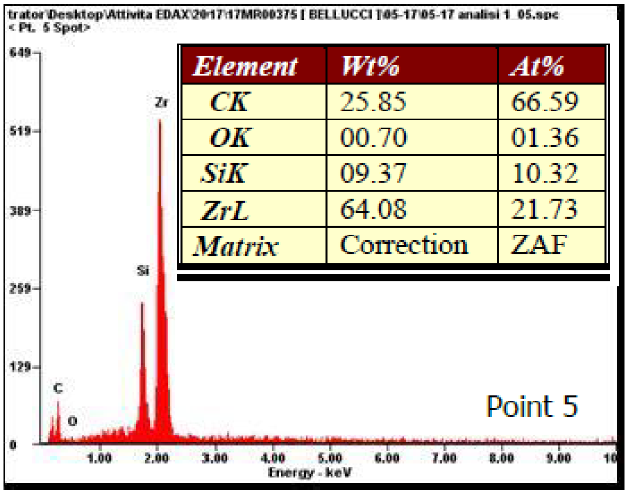

Element	Wt%	At%
CK	25.85	66.59
OK	00.70	01.36
SiK	09.37	10.32
ZrL	64.08	21.73
Matrix	Correction	ZAF

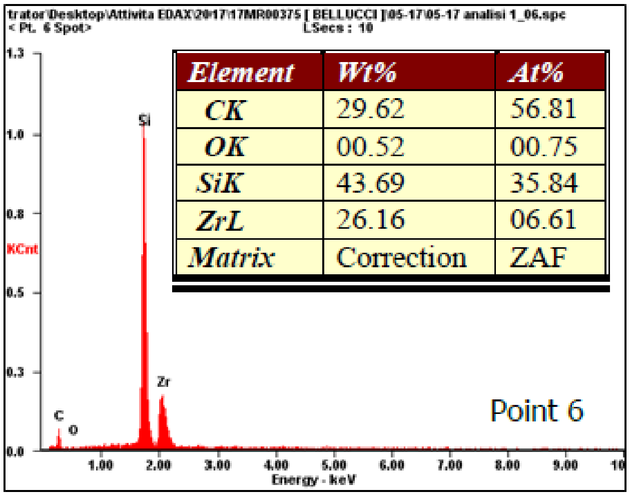

Element	Wt%	At%
CK	29.62	56.81
OK	00.52	00.75
SiK	43.69	35.84
ZrL	26.16	06.61
Matrix	Correction	ZAF

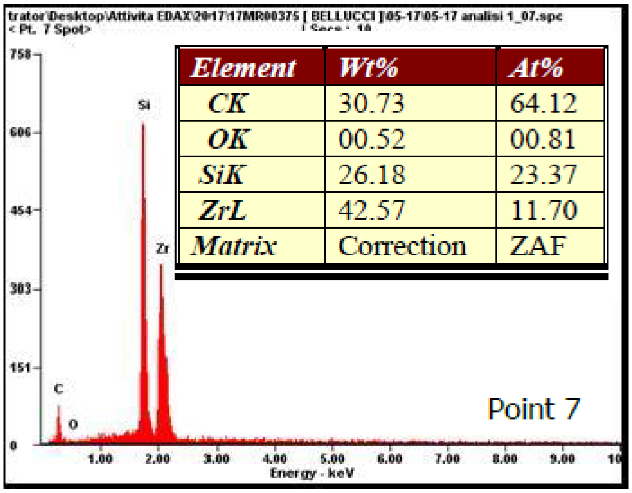

Element	Wt%	At%
CK	30.73	64.12
OK	00.52	00.81
SiK	26.18	23.37
ZrL	42.57	11.70
Matrix	Correction	ZAF

Figure S7. SEM-EDS analysis of SEM-EDS analysis of the coating B2, ZrB2-SiC (50%-50%): point analysis.

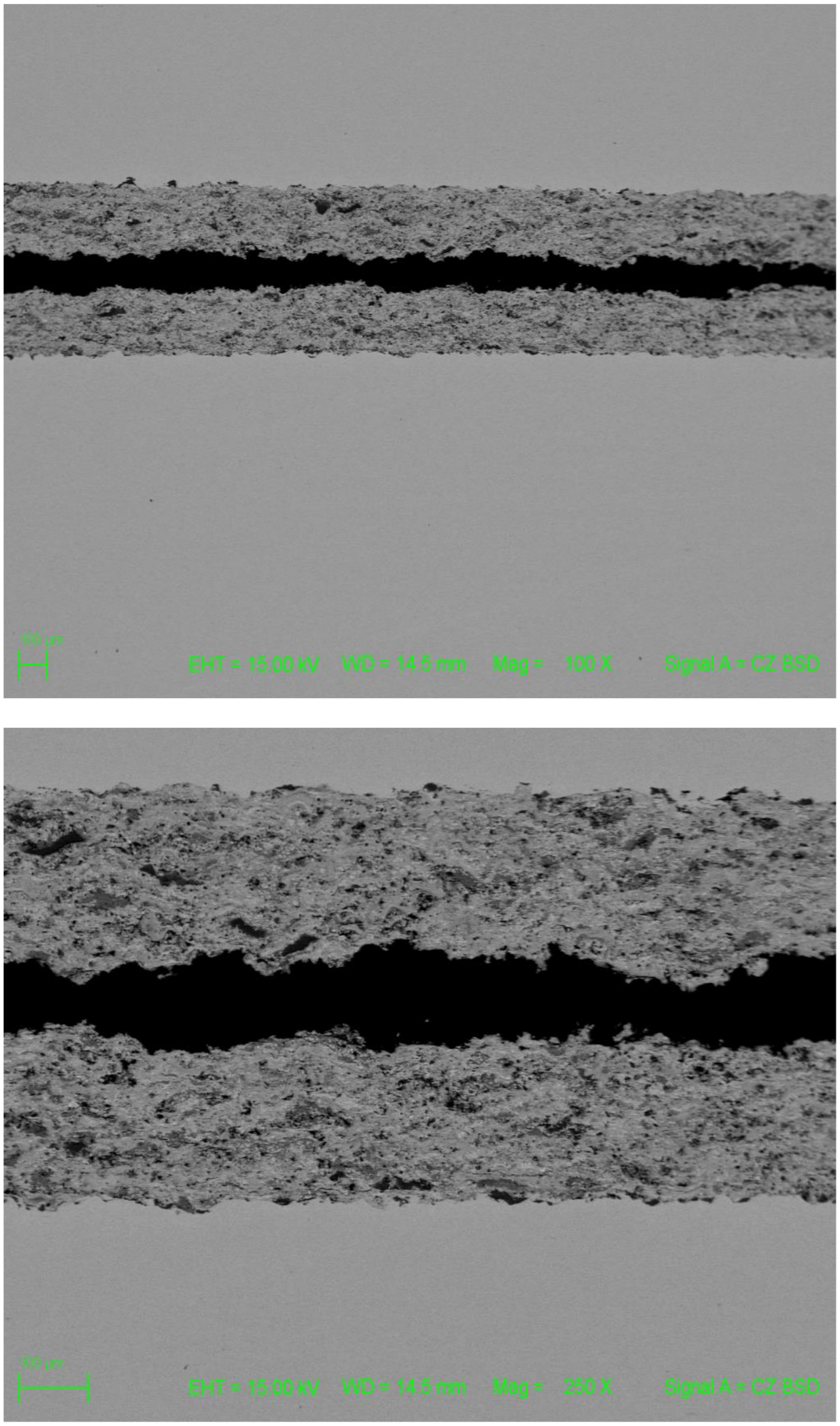

Figure S8. SEM image of the coating B1, ZrB_2-SiC (70%-30%)—metallographic cross section: two halves of the coating cross section are compared (black area = resin).

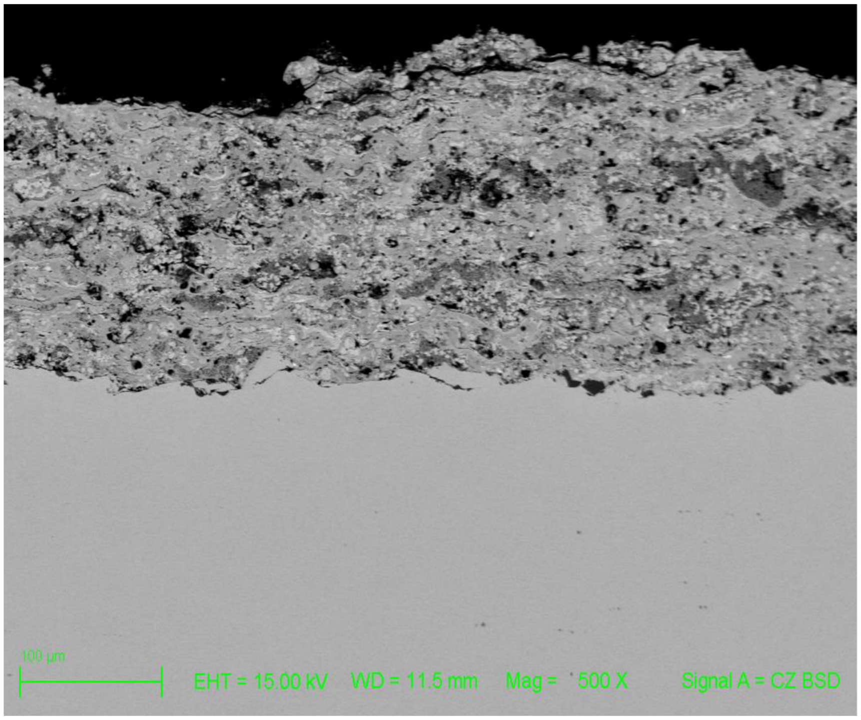

Figure S9. SEM image of the coating B1, ZrB2-SiC (70%-30%)—metallographic cross section: details.

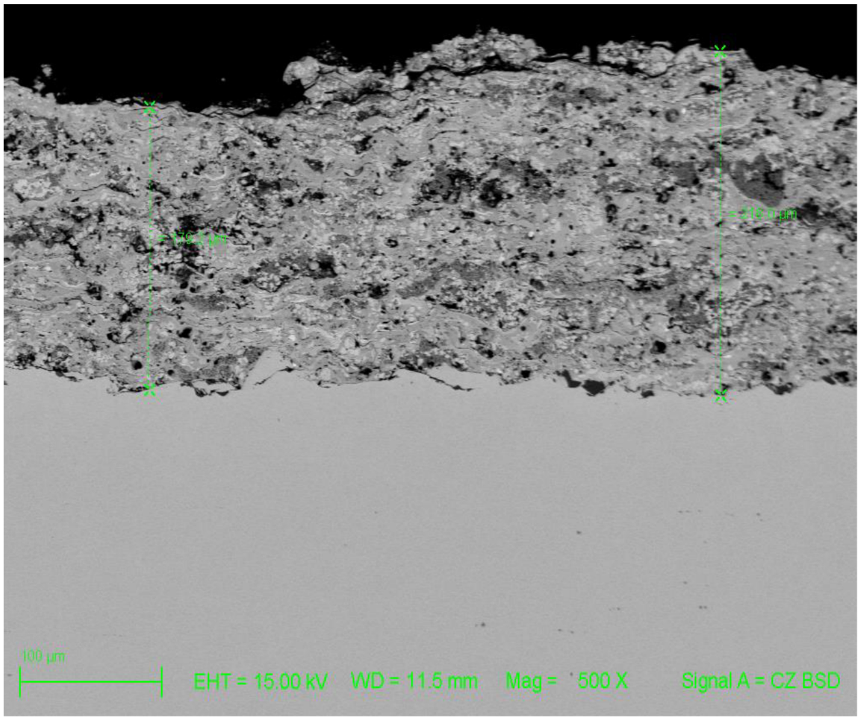

Figure S10. SEM image of the coating B1, ZrB2-SiC (70%-30%)—metallographic cross section: coating thickness.

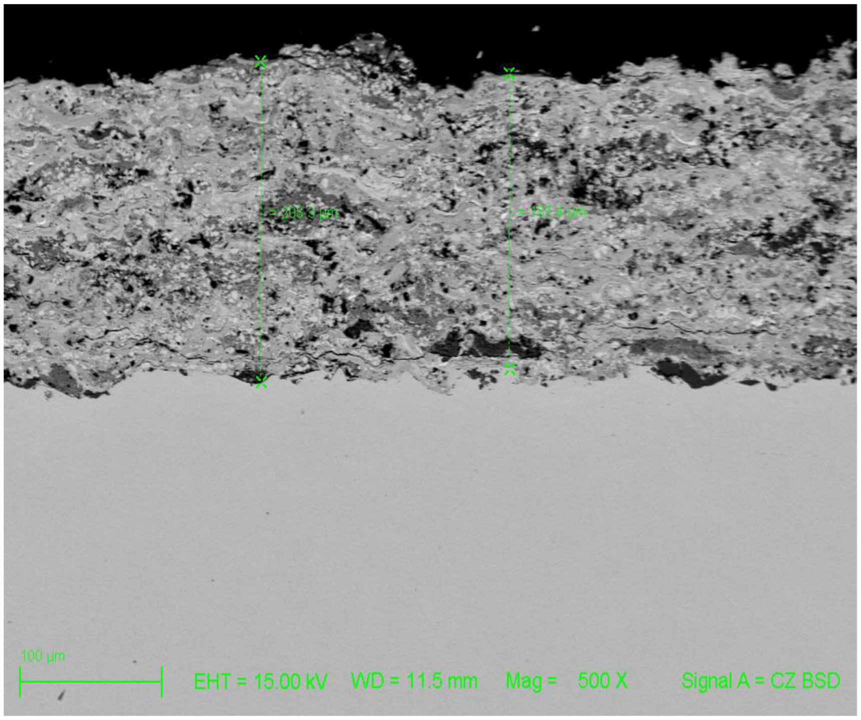

Figure S11. SEM image of the coating B1, ZrB2-SiC (70%-30%)—metallographic cross section: coating thickness.

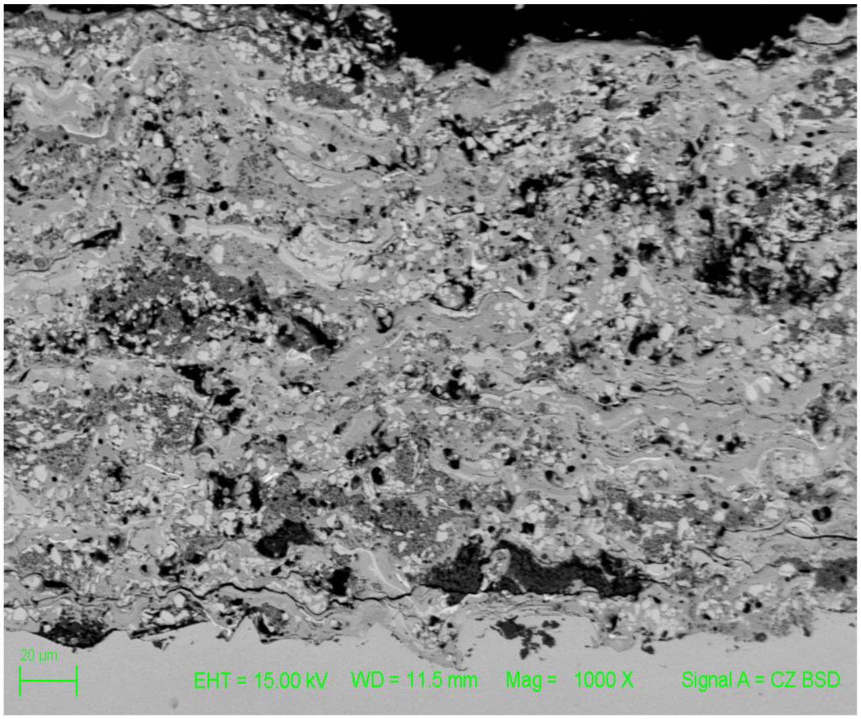

Figure S12. SEM image of the coating B1, ZrB2-SiC (70%-30%)—metallographic cross section: details.

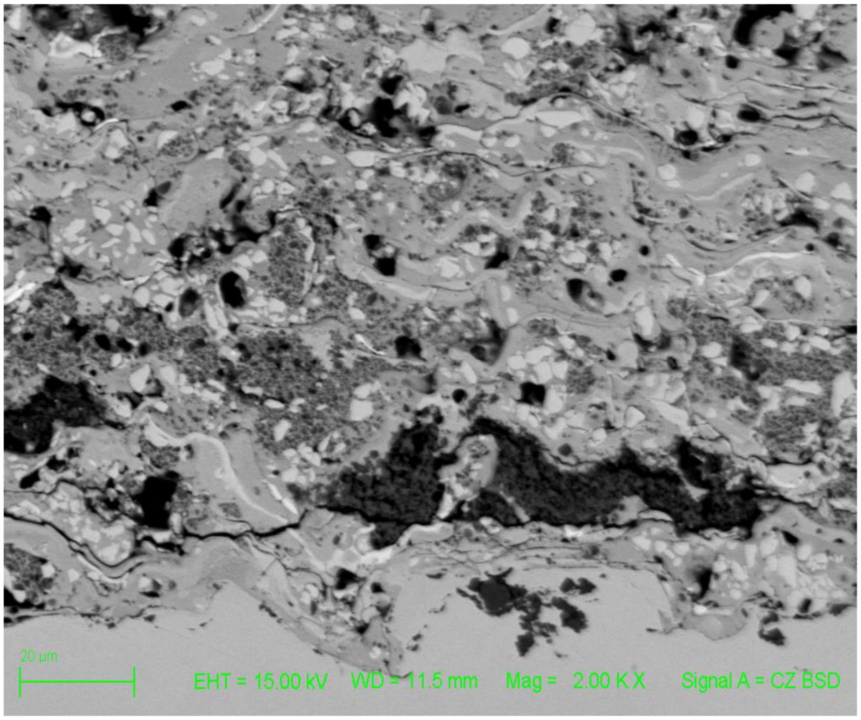

Figure S13. SEM image of the coating B1, ZrB2-SiC (70%-30%)—metallographic cross section: details.

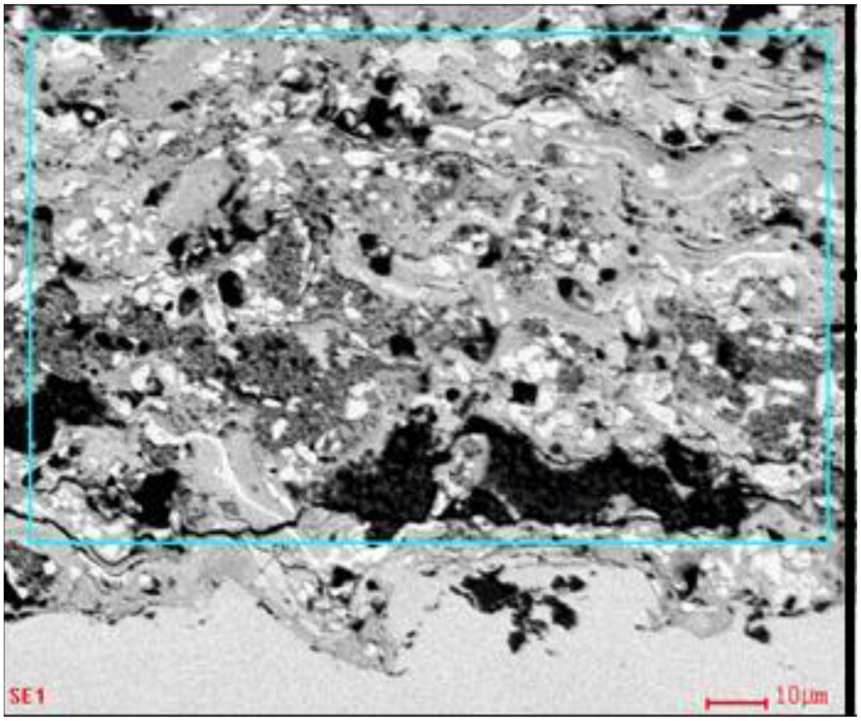

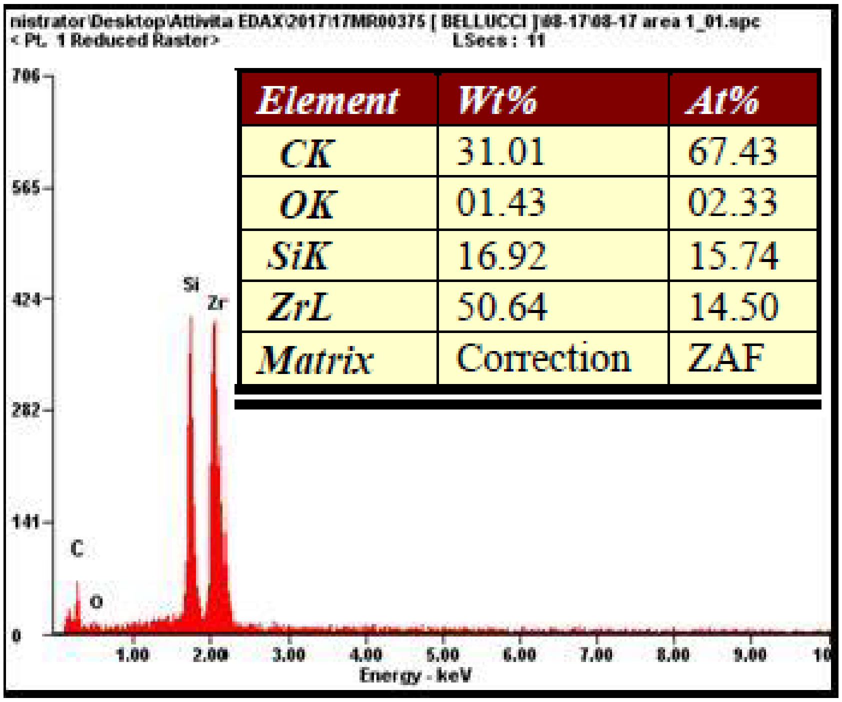

Figure S14. SEM-EDS analysis of the coating B1, ZrB2-SiC (70%-30%): area analysis.

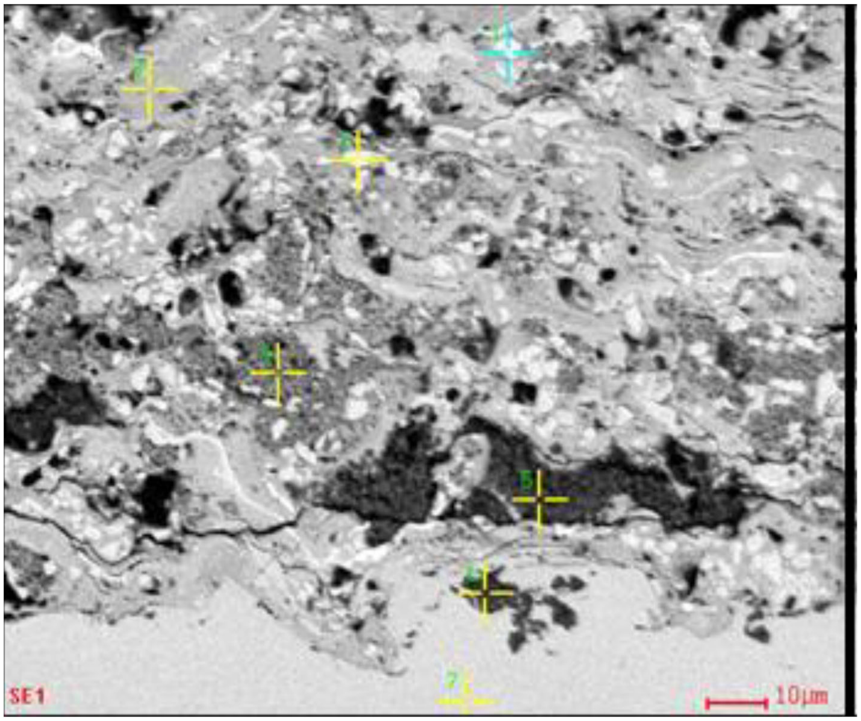

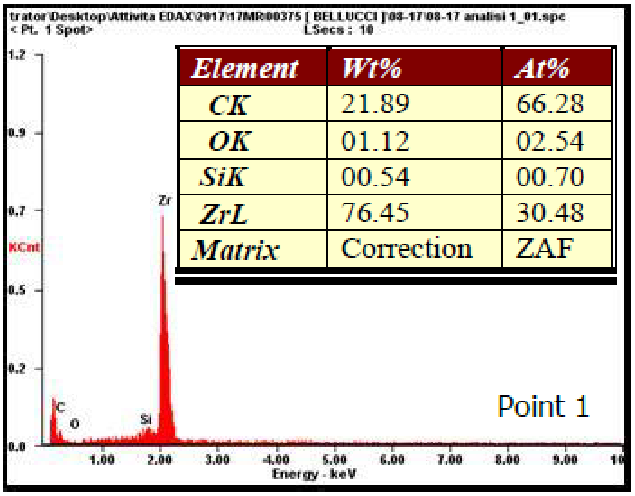
< Pt. 1 Spot>
LSecs : 10
Element | Wt% | At%
CK | 21.89 | 66.28
OK | 01.12 | 02.54
SiK | 00.54 | 00.70
ZrL | 76.45 | 30.48
Matrix | Correction | ZAF
KCnt
C
O
Si
Zr
Point 1
Energy - keV

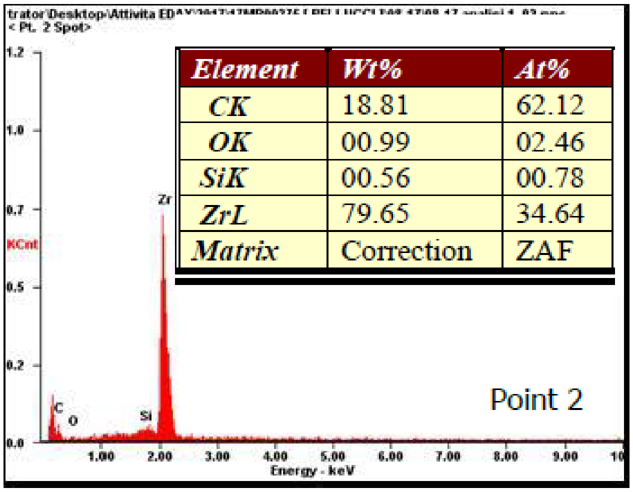
< Pt. 2 Spot>
Element | Wt% | At%
CK | 18.81 | 62.12
OK | 00.99 | 02.46
SiK | 00.56 | 00.78
ZrL | 79.65 | 34.64
Matrix | Correction | ZAF
KCnt
C
O
Si
Zr
Point 2
Energy - keV

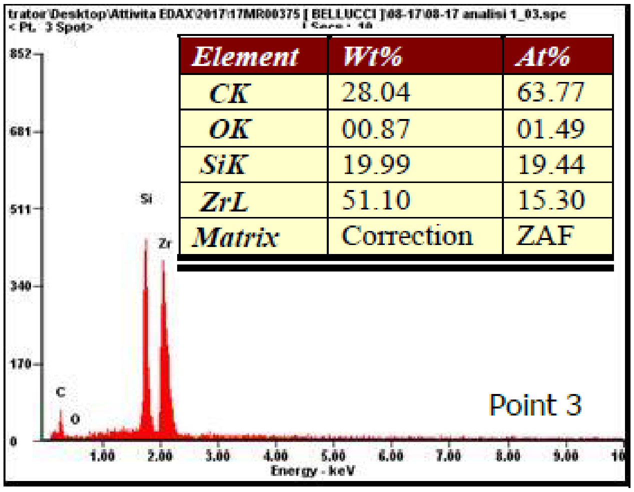
< Pt. 3 Spot>
Element | Wt% | At%
CK | 28.04 | 63.77
OK | 00.87 | 01.49
SiK | 19.99 | 19.44
ZrL | 51.10 | 15.30
Matrix | Correction | ZAF
C
O
Si
Zr
Point 3
Energy - keV

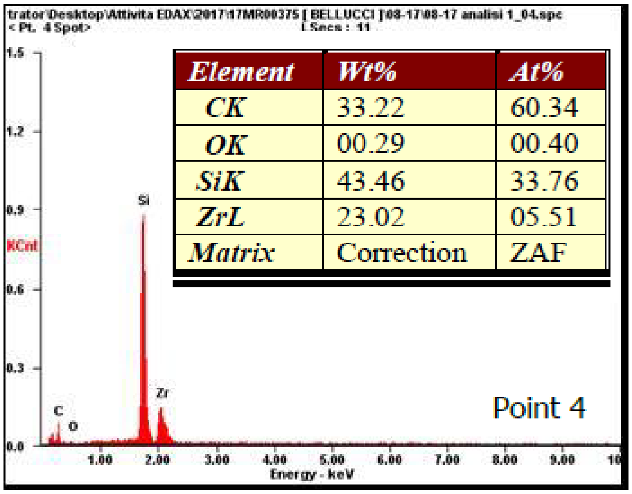

Element	Wt%	At%
CK	33.22	60.34
OK	00.29	00.40
SiK	43.46	33.76
ZrL	23.02	05.51
Matrix	Correction	ZAF

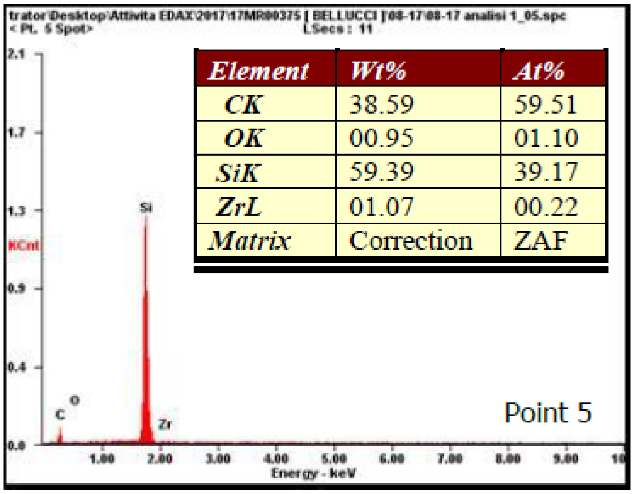

Element	Wt%	At%
CK	38.59	59.51
OK	00.95	01.10
SiK	59.39	39.17
ZrL	01.07	00.22
Matrix	Correction	ZAF

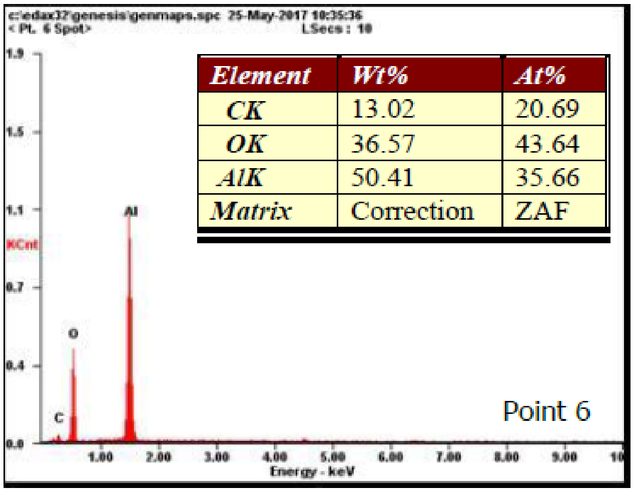

Element	Wt%	At%
CK	13.02	20.69
OK	36.57	43.64
AlK	50.41	35.66
Matrix	Correction	ZAF

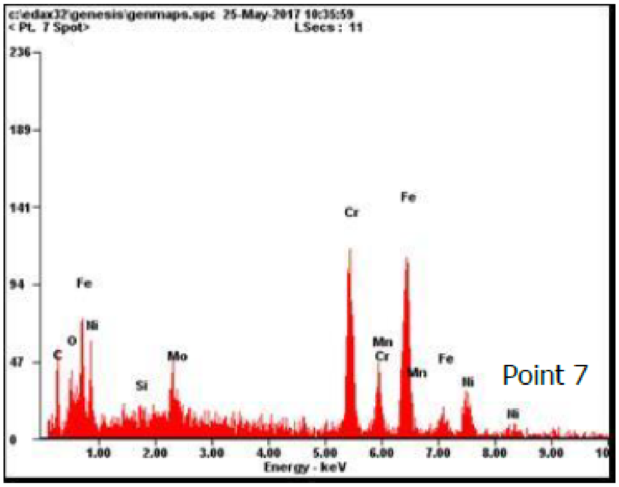

Element	*Wt%*	*At%*
CK	10.50	34.03
OK	01.96	04.77
SiK	00.58	00.80
MoL	03.29	01.34
CrK	22.80	17.08
MnK	06.30	04.47
FeK	39.10	27.26
NiK	15.47	10.26
Matrix	Correction	ZAF

Figure S15. SEM-EDS analysis of SEM-EDS analysis of the coating B1, ZrB2-SiC (70%-30%): point analysis.
